# Broken Arrows: Hardy–Unruh Chains and Quantum Contextuality

**DOI:** 10.3390/e25121568

**Published:** 2023-11-21

**Authors:** Michael Janas, Michel Janssen

**Affiliations:** School of Physics and Astronomy, University of Minnesota, Minneapolis, MN 55455, USA; jana0030@umn.edu

**Keywords:** Hardy paradox, quantum contextuality, correlation polytopes, Bell inequalities

## Abstract

Hardy and Unruh constructed a family of non-maximally entangled states of pairs of particles giving rise to correlations that cannot be accounted for with a local hidden-variable theory. Rather than pointing to violations of some Bell inequality, however, they pointed to apparent clashes with the basic rules of logic. Specifically, they constructed these states and the associated measurement settings in such a way that the outcomes satisfy some conditionals but not an additional one entailed by them. Quantum mechanics avoids the broken ‘if …then …’ arrows in such Hardy–Unruh chains, as we call them, because it cannot simultaneously assign truth values to all conditionals involved. Measurements to determine the truth value of some preclude measurements to determine the truth value of others. Hardy–Unruh chains thus nicely illustrate quantum contextuality: which variables do and do not obtain definite values depends on what measurements we decide to perform. Using a framework inspired by Bub and Pitowsky and developed in our book *Understanding Quantum Raffles* (co-authored with Michael E. Cuffaro), we construct and analyze Hardy–Unruh chains in terms of fictitious bananas mimicking the behavior of spin-$\frac{1}{2}$ particles.

## 1. Introduction

The standard way to show that quantum mechanics allows correlations impossible in classical (more precisely: local hidden-variable) theories is to point to violations of some Bell inequality. The classic example is the violation of the Clauser–Horne–Shimony–Holt (CHSH) inequality [1] by correlations between the outcomes of certain measurements on pairs of photons in a maximally entangled state. An alternative approach is to show that quantum mechanics allows correlations that seem to clash with basic logic. The work by Hardy [2,3] and Unruh [4] that we examine in this paper provides intriguing examples of this approach (the most famous example, undoubtedly, is due to Greenberger, Horne and Zeilinger [5]). In this approach, at least in principle, one combination of measurement outcomes suffices to rule out a local hidden-variable theory for the relevant quantum correlations, whereas in the more familiar approach, we need to consider the statistics of many outcomes.

Hardy [2,3] constructed a family of non-maximally entangled two-particle states and concomitant measurement settings such that the measurement outcomes satisfy two conditionals, but not a third, which would seem to be a direct consequence of the first two. Schematically,

(1) A→C,B→D,(A&B)↛(C&D).

This is what is known as Hardy’s paradox.

Inspired by Hardy, Unruh [4] constructed a family of states and settings such that the outcomes satisfy three conditionals, but not a fourth, which would seem to follow directly from the first three on the basis of the transitivity of the ‘if … then’ relation. Schematically,

(2) A→B,B→C,C→D,A↛D.

Such broken ‘if … then …’ arrows are allowed in quantum mechanics for the same reason that violations of Bell inequalities are. Local hidden-variable theories simultaneously assign truth values to propositions *A*, *B*, *C* and *D* above. Quantum mechanics does not. To assign truth values to all four propositions, one would simultaneously have to measure observables represented by non-commuting operators. These Hardy–Unruh chains of conditionals—as we call the sets of conditionals in Equations (1) and (2)—thus illustrate quantum contextuality: which observables do and do not obtain definite values depends on what measurements we decide to perform.

In this paper, we use the framework inspired by Bub [6] and Pitowsky [7] and developed by Janas, Cuffaro and Janssen [8] to construct and analyze these Hardy–Unruh chains. In Section 2, we review the elements we need from our book. In Section 3 and Section 4, we construct the states and measurement settings giving rise to the broken arrows in Equations (1) and (2). In Section 5, we examine the relation between these broken arrows and violations of the relevant Bell inequality, which, as we will see, is a special case of the CHSH inequality. On the basis of this analysis, we conclude, in Section 6, that broken arrows and violations of Bell inequalities are different but ultimately equivalent ways of bringing out quantum contextuality.^1^

## 2. Preliminaries

In our book, *Understanding Quantum Raffles* [8], inspired by Bub’s *Bananaworld* [6], we used the imagery of peeling and tasting fictitious bananas mimicking the measurement of spin components of (half-)integer spin particles. We modified Bub’s banana-peeling scheme to tighten the analogy between our bananas and particles with spin. In this paper, as in most of our book, we focus on bananas mimicking the behavior of spin-$\frac{1}{2}$ particles.^2^

Imagine picking a pair of such bananas, connected at the stem, from a particular species of banana tree, breaking them apart and giving one to Alice and one to Bob. Alice and Bob then choose a peeling direction, i.e., a direction in which they are required to hold their banana while peeling it. When finished peeling, they take a bite to determine whether their banana tastes yummy or nasty. It is a key feature of our banana imagery that, when the bananas are still on the banana tree, they do not possess a specific taste nor any properties predetermining their taste upon being peeled and tasted. They somehow only acquire their taste upon being peeled and tasted. Yummy and nasty are the only possible values for taste for this species of banana.

Readers put off by our *Bananaworld* imagery can replace (i) bananas with spin-$\frac{1}{2}$ particles; (ii) species of banana trees with states in which we prepare pairs of such particles (though we will also talk about pairs of bananas in particular quantum states); (iii) peeling directions (or peelings for short) with orientations of Du Bois (or Stern–Gerlach) magnets; (iv) the actual peeling with sending particles through a Du Bois magnet; (v) tasting with having a particle hit a screen with a photo-emulsion behind the magnet; and (vi) yummy and nasty with spin up and spin down, respectively. Our fictitious bananas, however, are not just a gimmick. They also underscore that the correlations examined in our book and in this paper can be realized in many different physical systems.^3^

**Figure 1:**
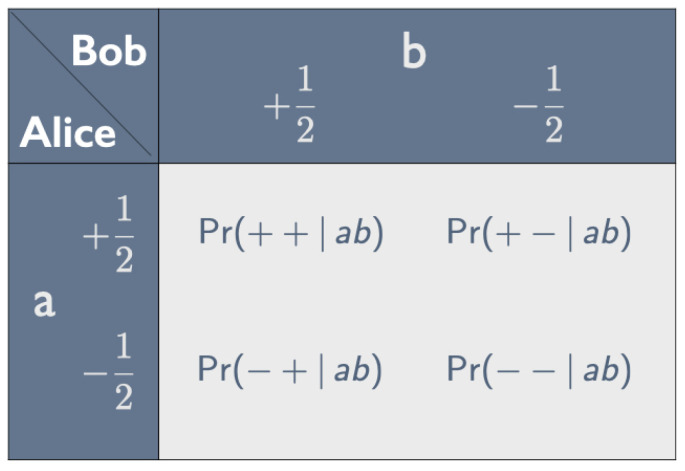
Correlation array for Alice peeling *a* and Bob peeling *b*.

Suppose Alice peels *a* and Bob peels *b*. The correlations between the tastes they find, which persist no matter how far apart they are, can be represented by a *correlation array* (see Figure 1). In analogy with the values $+\frac{1}{2}\hbar$ and $-\frac{1}{2}\hbar$ for spin up and spin down (where *ℏ* is Planck’s constant divided by 2π), we assign the numerical values $+\frac{1}{2}$ and $-\frac{1}{2}$ to the tastes yummy and nasty in some appropriate units. Unless we need these values to calculate expectation values, we simply use + for yummy and − for nasty. The four entries in the correlation array give the probabilities of the four possible outcomes for this combination of peelings.

For now, we restrict our attention to species of banana trees (but this does *not* include the species giving rise to Hardy–Unruh chains) on which bananas grow in pairs such that the correlations between their tastes have two special properties:

1. No matter what peelings Alice and Bob use, the probability of them finding yummy or nasty is always $\frac{1}{2}$.
2. If Alice and Bob use the same peeling, they always find opposite tastes.

**Figure 2:**
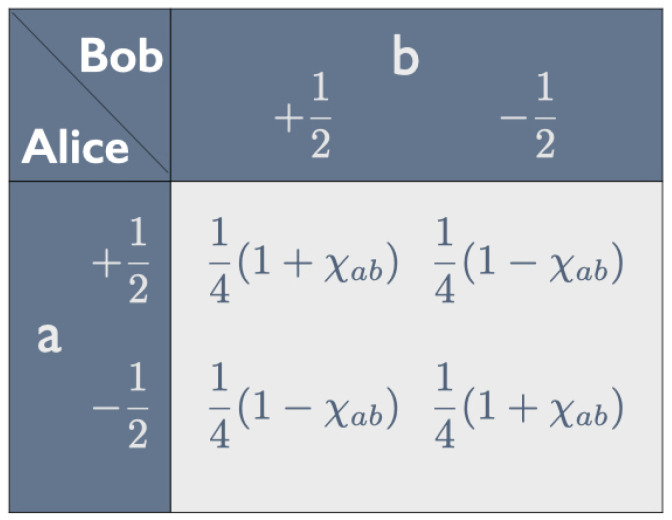
Parametrization of correlation array in Figure 1 given property 1.

Property 1 means that the entries in both rows and both columns of the correlation array in Figure 1 add up to $\frac{1}{2}$. In that case, as shown in Figure 2, the correlation array can be fully characterized by the parameter $-1\leq \chi_{ab}\leq +1$, with $\chi_{ab}=-1$ if the peelings *a* and *b* are the same (property 2).

**Figure 3:**
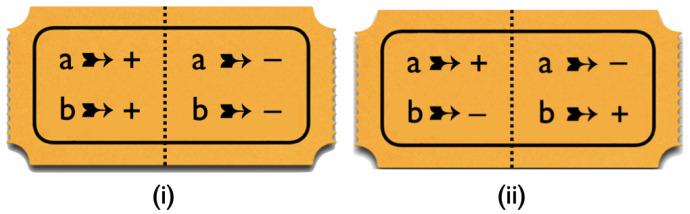
Raffle tickets for the simulation of the correlation array in Figure 2.

We can simulate these correlations for any value of $\chi_{ab}$ with the kind of raffle introduced in our book ([8], Section 2.5) as a model for local hidden-variable theories. In this case, the raffle consists of a basket with a mix of the two types of tickets shown in Figure 3, with the tastes of both bananas for both peelings printed on them. We draw tickets from this basket, tear them in half along the perforation indicated by the dashed line, and randomly give one half to Alice and one half to Bob. That the values for *a* and *b* on the two sides of the ticket are opposite takes care of property 2. That we randomly decide which half goes to Alice and which half to Bob takes care of property 1.

A raffle that exclusively has tickets of type (i) will give a perfect anti-correlation between Alice’s result for *a* and Bob’s result for *b*. In that case, the entries on the diagonal in Figure 1 and Figure 2 are 0, while the off-diagonal ones are $\frac{1}{2}$. Thus, for tickets of type (i), $\chi_{ab}=-1$. A raffle that exclusively has tickets of type (ii) will give a perfect correlation. In that case, the off-diagonal entries in Figure 1 and Figure 2 are 0 and those on the diagonal are $\frac{1}{2}$. Thus, for tickets of type (i), $\chi_{ab}=1$. To simulate the correlation in Figure 2 for arbitrary values of $\chi_{ab}$, we need a raffle with a fraction $\frac{1}{2}\left(1-\chi_{ab}\right)$ of type-(i) tickets and a fraction $\frac{1}{2}\left(1+\chi_{ab}\right)$ of type-(ii) tickets.

It turns out that, for all values between −1 and +1, $\chi_{ab}$ is the (*Pearson*) *correlation coefficient* of the variables $A_{a}$ and $B_{b}$, the taste Alice finds when peeling *a* and the taste Bob finds when peeling *b*.^4^ The correlation coefficient of two stochastic variables *X* and *Y* is defined as the covariance, Cov(XY)≡〈(X−〈X〉)(Y−〈Y〉)〉, divided by the standard deviations, $\sigma_{X}$ and $\sigma_{Y}$, the square roots of the variances, $\langle {\left(X-\langle X\rangle \right)}^{2}\rangle$ and $\langle {\left(Y-\langle Y\rangle \right)}^{2}\rangle$. What simplifies matters in the case of the variables $A_{a}$ and $B_{b}$ is that they are *balanced*, i.e., their two possible values are each other’s opposite and these two values are equiprobable ([8], p. 68). This means that their expectation values, $\langle A_{a}\rangle$ and $\langle B_{b}\rangle$, vanish and that the correlation coefficient is given by

(3) $$\rho_{A_{a}B_{b}}=\frac{\langle A_{a}B_{b}\rangle }{\sqrt{\langle A_{a}^{2}\rangle }\sqrt{\langle B_{b}^{2}\rangle }}.$$

Inspection of the correlation arrays in Figure 1 and Figure 2 tells us that

(4) $$\begin{array}{ccc} \langle A_{a}B_{b}\rangle & \;\;\;=\;\;\; & \frac{1}{4}\left(\mathrm{Pr}(+\;+\;|ab)+\mathrm{Pr}(-\;-\;|ab)\right)\;-\;\frac{1}{4}\left(\mathrm{Pr}(+\;-\;|ab)+\mathrm{Pr}(-\;+\;|ab)\right)\\ & \;\;\;=\;\;\; & \frac{1}{4}\cdot \frac{1}{2}\left(1+\chi_{ab}\right)\;-\;\frac{1}{4}\cdot \frac{1}{2}\left(1-\chi_{ab}\right)=\frac{1}{4}\;\chi_{ab}; \end{array}$$

that

(5) $$\langle A_{a}^{2}\rangle =\frac{1}{4}\left(\mathrm{Pr}(+\;+\;|ab)+\mathrm{Pr}(+\;-\;|ab)\right)+\frac{1}{4}\left(\mathrm{Pr}(-\;+\;|ab)+\mathrm{Pr}(-\;-\;|ab)\right)=\frac{1}{4};$$

and that, similarly, $\langle B_{b}^{2}\rangle =\frac{1}{4}$. Substituting these results into Equation (3), we see that the correlation coefficient is indeed equal to the parameter characterizing the correlation in Figure 2:

(6) $$\rho_{A_{a}B_{b}}=\frac{\frac{1}{4}\;\chi_{ab}}{\frac{1}{2}\cdot \frac{1}{2}}=\chi_{ab}.$$

As noted above, unless $\chi_{ab}=\pm 1$, we need a mix of tickets to simulate the correlation array in Figure 2 with one of our raffles. With our quantum bananas, as we will show below, we can produce this correlation array for arbitrary values $-1<\chi_{ab}<1$ with pairs of bananas in the familiar fully entangled singlet state, *but with different choices for the peeling directions a and b*.

Using the bases ${\{|\pm \rangle }_{a}\}$ and ${\{|\pm \rangle }_{b}\}$ of eigenvectors of the operators representing the observables ‘taste when peeled in the *a*-direction’ and ‘taste when peeled in the *b*-direction’ for the one-banana Hilbert space to construct bases for the two-banana Hilbert space, we can write the singlet state as:

(7) $$|\psi_{\mathrm{singlet}}\rangle =\frac{1}{\sqrt{2}}\left({|+-\rangle }_{aa}-{|-+\rangle }_{aa}\right)=\frac{1}{\sqrt{2}}\left({|+-\rangle }_{bb}-{|-+\rangle }_{bb}\right),$$

where ${|+-\rangle }_{aa}$, etc., is shorthand for the tensor product ${|+\rangle }_{a}⊗{|-\rangle }_{a}$, etc.

**Figure 4:**
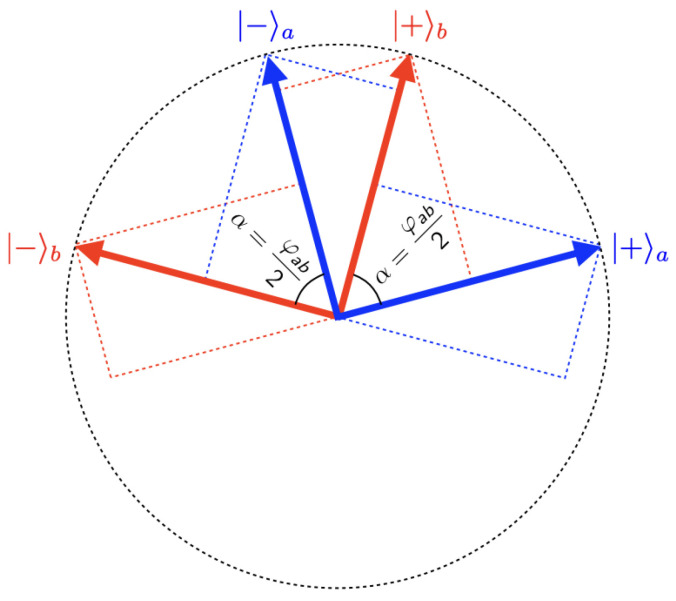
Eigenvectors for ‘taste when peeled in the *a*-direction’ and ‘taste when peeled in the *b*-direction’ in the one-banana Hilbert space, where $\varphi_{ab}=2\alpha$ is the angle between the peeling directions *a* and *b*.

The relation between the *a*-basis and the *b*-basis is illustrated in Figure 4. The angle α between these pairs of eigenvectors is equal to half the angle $\varphi_{ab}$ between the peeling directions *a* and *b*. The transformation from the *b*-basis to the *a*-basis is given by:

(8) $$\begin{array}{c} {|+\rangle }_{a}={\cos\alpha \;|+\rangle }_{b}-\sin\alpha \;{|-\rangle }_{b},\\ {|-\rangle }_{a}={\sin\alpha \;|+\rangle }_{b}+\cos\alpha \;{|-\rangle }_{b}\;; \end{array}$$

its inverse by:

(9) $$\begin{array}{c} {|+\rangle }_{b}={\cos\alpha \;|+\rangle }_{a}+\sin\alpha \;{|-\rangle }_{a},\\ {|-\rangle }_{b}={-\sin\alpha \;|+\rangle }_{a}+\cos\alpha \;{|-\rangle }_{a}. \end{array}$$

To find the probabilities of the various combinations of tastes when Alice peels *a* and Bob peels *b*, we use these transformation equations to write the singlet state in the ab-basis:

(10) $$|\psi_{\mathrm{singlet}}\rangle =\frac{1}{\sqrt{2}}\left(\sin\alpha {|++\rangle }_{ab}\;+\;\cos\alpha {|+-\rangle }_{ab}\;-\;\cos\alpha {|-+\rangle }_{ab}\;+\;\sin\alpha {|--\rangle }_{ab}\right).$$

**Figure 5:**
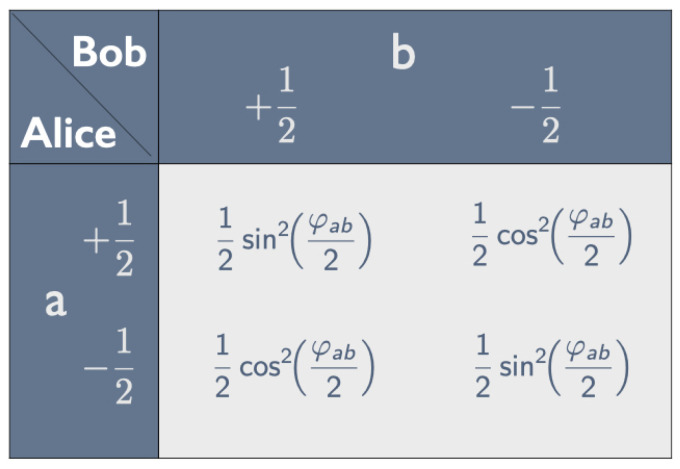
Correlation array for taste-and-peel experiment with bananas in the singlet state.

The Born rule tells us that the probabilities of finding the various combinations of tastes for this combination of peelings are given by the squares of the coefficients of the corresponding terms of the singlet state in this basis. Recalling that $\alpha =\varphi_{ab}/2$, we thus arrive at the correlation array in Figure 5.

Using this correlation array to calculate the correlation coefficient (see Equation (3)), we find:

(11) $$\rho_{A_{a}B_{b}}=\frac{\frac{1}{4}\cdot \sin^{2}\;\left(\frac{\varphi_{ab}}{2}\right)-\frac{1}{4}\cdot \cos^{2}\;\left(\frac{\varphi_{ab}}{2}\right)}{\frac{1}{2}\cdot \frac{1}{2}}=-\cos\varphi_{ab}.$$

We saw earlier (see Equation (6)) that $\rho_{A_{a}B_{b}}$ is equal to the parameter $\chi_{ab}$ characterizing the correlation array in Figure 2. With the appropriate choice of peeling directions, we can thus obtain this correlation array for any value $-1\leq \chi_{ab}\leq 1$ with the appropriate measurements on the same quantum state, whereas we needed a mix of tickets to obtain this correlation array with one of our raffles.

In *Understanding Quantum Raffles* [8], we used the tools introduced above to analyze the correlations found in an experimental setup due to Mermin [10] in which Alice and Bob peel and taste bananas in the singlet state choosing between three different peeling directions, *a*, *b* and *c*. The correlations between the tastes found by Alice and Bob in this Mermin setup can be represented by a 3×3 correlation array with cells of the form shown in Figure 2 with $\chi_{ab}=-\cos\varphi_{ab}$, etc. (see Figure 5 and Equation (11)).

Because of the symmetry of the singlet state, the cells of the correlation array on one side of the diagonal (ab, ac and bc) are the same as those on the other side (ba, ca and cb). In the cells on the diagonal, we have a perfect anti-correlation (if a=b, $\varphi_{ab}=0$ and $\chi_{ab}=-1$). A correlation array for this Mermin setup can thus be characterized by the correlation coefficients for half of its off-diagonal cells, $\chi_{ab}$, $\chi_{ac}$ and $\chi_{bc}$, with all three taking on values between −1 and +1.

Inspired by Pitowsky [7], we used these coefficients as coordinates of a point in a 2×2×2 cube, the *non-signaling polytope* (P) for the Mermin setup (see Figure 7). The part of P allowed by quantum mechanics is called the *quantum convex set* (Q); the part allowed by local hidden-variable theories the *local polytope* (L).^5^

**Figure 6:**
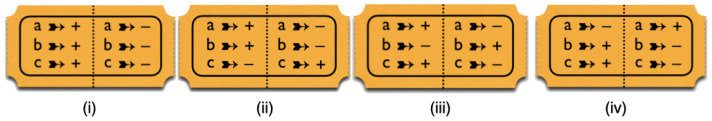
Tickets for a raffle meant to simulate the correlation array for the Mermin setup.

We derive the inequalities defining L and Q in this case. As our model for a local hidden-variable theory, we use a raffle with a mix of the four types of tickets shown in Figure 6.

The values of the correlation coefficients for raffles with only one type of ticket can be read directly off that ticket. For example, if the values for *a* and *b* on opposite sides of the ticket are the same, $\chi_{ab}=1$; if they are opposite, $\chi_{ab}=-1$. Table 1 collects the values of $\chi_{ab}$, $\chi_{ac}$ and $\chi_{bc}$ for ticket types (i)–(iv).

**Figure 7:**
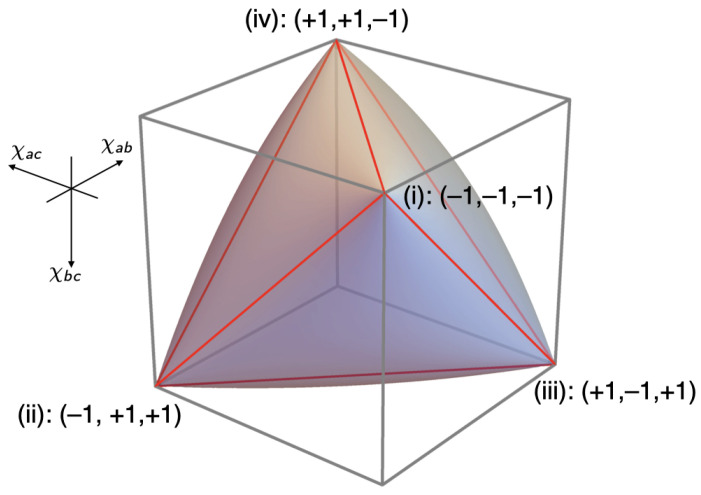
The non-signaling polytope (P), the quantum convex set (Q) and the local polytope (L) for the Mermin setup.

The correlations produced by raffles with just one of these four ticket-types are represented by the vertices that are labeled (i) through (iv) in the non-signaling cube in Figure 7. The *local polytope* (L) for the Mermin setup is the tetrahedron formed by these four vertices.

The Bell inequality for the Mermin setup corresponds to one of the four facets of the tetrahedron, the one with the vertices (ii), (iii) and (iv). The pair of inequalities associated with this facet, which can be read off Table 1, is:

(12) $$-3\leq \chi_{ab}+\chi_{ac}+\chi_{bc}\leq 1.$$

This is the direct analogue of the CHSH inequality, the Bell inequality for a setup involving four rather than three different peelings, with Alice peeling *a* or *b* and Bob peeling $a^{\prime }$ or $b^{\prime }$ (cf. Equation (48) below and Chapter 5 in our book [8]). To fully characterize the local polytope for the Mermin setup, we need three more pairs of inequalities such as the ones in Equation (12), corresponding to the other three facets of the tetrahedron in Figure 7.

To find the *quantum convex set* (Q) for the Mermin setup, we consider the 3×3 matrix formed by the correlation coefficients characterizing the nine cells of its correlation array. Using that $\chi_{ab}=-\cos\varphi_{ab}=-{\vec{e}}_{a}\cdot {\vec{e}}_{b}$, etc. (where ${\vec{e}}_{a}$ and ${\vec{e}}_{b}$ are unit vectors in the peeling directions *a* and *b*), we can write this *correlation matrix* as:

(13) $$\chi \equiv \left(\begin{array}{ccc} \;-1\; & \;\chi_{ab}\; & \;\chi_{ac}\;\\ \;\chi_{ba} & \;-1\; & \;\chi_{bc}\;\\ \;\chi_{ca}\; & \;\chi_{cb}\; & \;-1 \end{array}\right)=-\left(\begin{array}{ccc} \;{\vec{e}}_{a}\cdot {\vec{e}}_{a}\; & \;{\vec{e}}_{a}\cdot {\vec{e}}_{b}\; & \;{\vec{e}}_{a}\cdot {\vec{e}}_{c}\;\\ \;{\vec{e}}_{b}\cdot {\vec{e}}_{a} & \;{\vec{e}}_{b}\cdot {\vec{e}}_{b}\; & \;{\vec{e}}_{b}\cdot {\vec{e}}_{c}\;\\ \;{\vec{e}}_{c}\cdot {\vec{e}}_{a}\; & \;{\vec{e}}_{c}\cdot {\vec{e}}_{b}\; & \;{\vec{e}}_{c}\cdot {\vec{e}}_{c} \end{array}\right).$$

This is (minus) a *Gram matrix*, which has the property that its determinant cannot be negative: −detχ≥0. This gives us the constraint we are looking for:

(14) $$1-\chi_{ab}^{2}-\chi_{ac}^{2}-\chi_{bc}^{2}-2\;\chi_{ab}\;\chi_{ac}\;\chi_{bc}\geq 0.$$

This non-linear inequality defines the elliptope representing the quantum convex set (Q) for the Mermin setup in Figure 7. Taking a slice of this figure by setting one of the χ’s to zero, we obtain the Vitruvian-man-like cartoon in *Bananaworld* for P, Q and L in an arbitrary setup ([6], p. 107, Figure 5.2).

We now have all the ingredients we need from *Understanding Quantum Raffles* [8] to analyze the correlations found with Hardy and Hardy–Unruh states.

## 3. Hardy States

Hardy [2] cooked up a family of two-particle states, each member with its own combination of measurements to be performed on it, to illustrate the apparent breakdown of basic logic in quantum mechanics (see Equation (1)). We construct the states for a branch of this family in *Bananaworld*, in which Alice and Bob both use the same pair of peelings *a* and *b*. As we will see when we turn to the intimately related Hardy–Unruh family of states, other members of the Hardy family involve Alice and Bob using different pairs of peelings, which we will label (a,b) and $\left(a^{\prime },b^{\prime }\right)$, respectively.

### 3.1. Hardy Chain of Conditionals

Hardy states have four special properties that translate into corresponding properties of the correlations between the tastes found by Alice, peeling *a* or *b*, and Bob, peeling $a^{\prime }$ or $b^{\prime }$, which can but do not have to be the same as *a* and *b*.

1. There is no |+−〉 component in the $ba^{\prime }$-basis. Thus, if Alice peels *b* and finds +, then Bob will also find + when he peels $a^{\prime }$. Schematically: $A_{b_{+}}\to B_{a_{+}^{\prime }}$. Of course, this property of the state also implies $B_{a_{-}^{\prime }}\to A_{b_{-}}$, but this conditional is not part of the Hardy chain.
2. There is no |−+〉 component in the $ab^{\prime }$-basis. Thus, if Bob peels $b^{\prime }$ and finds +, then Alice will also find + when she peels *a*. Schematically: $B_{b_{+}^{\prime }}\to A_{a_{+}}$.
3. There is no |++〉 component in the $aa^{\prime }$-basis. Thus, if Alice peels *a* and Bob peels $a^{\prime }$, they cannot both find +. Schematically: we cannot have $A_{a_{+}}\&\;B_{a_{+}^{\prime }}$.
4. There is both a |++〉 and a |+−〉 component in the $bb^{\prime }$-basis. Thus, if Alice peels *b* and Bob peels $b^{\prime }$, it is possible for both of them to find +. Schematically: we can have $A_{b_{+}}$ and $B_{b_{+}^{\prime }}$.

**Figure 8:**
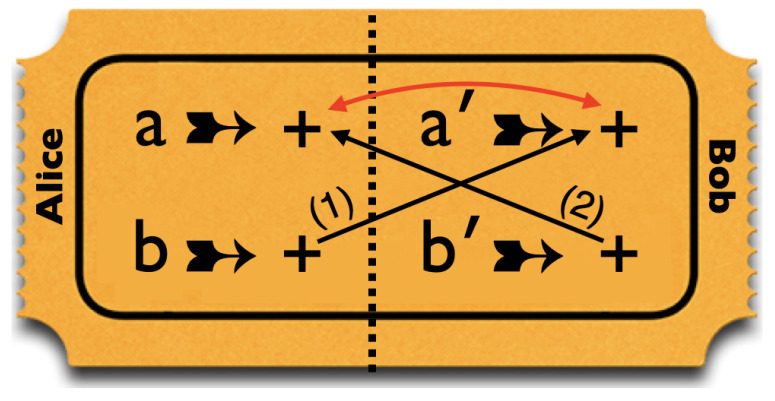
Conflicting demands on the design of a ticket for a raffle simulating the correlations found in measurements on Hardy states.

These four properties place contradictory demands on the design of tickets for a raffle simulating these correlations. This is illustrated in Figure 8. Since Alice and Bob use different pairs of peelings, the left side of the ticket always goes to Alice and the right side to Bob. Because of property 4, our raffle must contain *some* tickets with + for both *b* and $b^{\prime }$. Because of properties 1 and 2, such tickets must also have + for both *a* and $a^{\prime }$. However, because of property 3, our raffle is not allowed to contain *any* such tickets!

Following Hardy ([2], p. 1666), we can bring out the problem in a slightly different way (see also Kwiat and Hardy [16], p. 34). The conditionals $A_{b_{+}}\to B_{a_{+}^{\prime }}$ (property 1) and $B_{b_{+}^{\prime }}\to A_{a_{+}}$ (property 2) entail the composite conditional

(15) $$\left(A_{b_{+}}\;\mathrm{and}\;B_{b_{+}^{\prime }}\right)\to \left(A_{a_{+}}\;\mathrm{and}\;B_{a_{+}^{\prime }}\right).$$

However, this conditional is false: it is possible for the antecedent to be true (property 4) and the consequent to be false (property 3). Quantum mechanics avoids the broken arrow in Equation (15) by not allowing truth values to be assigned simultaneously to the antecedent and consequent. The same pair of bananas cannot be peeled and tasted twice: Alice cannot peel hers both *a* and *b*, and Bob cannot peel his both $a^{\prime }$ and $b^{\prime }$.

At this point, our banana imagery may suggest that we would run into the same problem with ordinary bananas, which, after all, can also only be peeled once. However, this is not the case. First of all, the taste of ordinary bananas does not depend on the direction in which they are held when peeled. Still, one could easily imagine bananas with this property. Their taste might depend, for instance, on their orientation with respect to the earth’s magnetic field when they are being peeled. Even for such bananas, however, we would expect the taste upon being peeled in whatever direction to be predetermined by some property that the banana already possesses before it is peeled. But the ticket in Figure 8 shows that, if these bananas have properties 1–4 above, their tastes cannot be predetermined. Thus, the explanation of how the Hardy chain of conditionals can be broken crucially hinges on contextuality and not just on the property, which our quantum bananas share with ordinary bananas, that they can only be peeled once.

### 3.2. Constructing Hardy States

We construct a branch of the family of Hardy states in *Bananaworld* with $a=a^{\prime }$ and $b=b^{\prime }$. Members of this branch can be labeled by the angle α, which is half the angle $\varphi_{ab}$ between the peeling directions *a* and *b*. The angle α thus runs from 0 to π/2. We start with property 3: the state has no |++〉 component in the aa-basis:

(16) $$|\psi_{H}\left(\alpha \right)\rangle =N\left(\alpha \right)\left(-\sin\alpha {|+-\rangle }_{aa}+\cos\alpha {|--\rangle }_{aa}-\sin\alpha {|-+\rangle }_{aa}\right),$$

where the factor

(17) $$N\left(\alpha \right)=\frac{1}{\sqrt{1+\sin^{2}\;\alpha }}$$

normalizes the state. The coefficients of the three components in the aa-basis were chosen with malice aforethought. Given our choice of peeling *b* to go with peeling *a*, these coefficients ensure that $|\psi_{H}\left(\alpha \right)\rangle$ also has properties 1 and 2. Combining the first and the second term on the right-hand side of Equation (16) and using Equation (9), the transformation from the *a*- to the *b*-basis, we can write $|\psi_{H}\left(\alpha \right)\rangle$ as

(18) $$\begin{array}{ccc} |\psi_{H}\left(\alpha \right)\rangle & \;\;\;=\;\;\; & N\left(\alpha \right)\left(\left\{{-\sin\alpha \;|+\rangle }_{a}+\cos\alpha \;{|-\rangle }_{a}\right\}⊗{|-\rangle }_{a}-\sin\alpha {|-+\rangle }_{aa}\right)\\ & \;\;\;=\;\;\; & N\left(\alpha \right)\left({|--\rangle }_{ba}-\sin\alpha {|-+\rangle }_{aa}\right), \end{array}$$

which shows that $|\psi_{H}\left(\alpha \right)\rangle$ has no |+−〉 component in the ba-basis (property 1). Combining the second and the third term on the right-hand side of Equation (16), we can also write $|\psi_{H}\left(\alpha \right)\rangle$ as

(19) $$\begin{array}{ccc} |\psi_{H}\left(\alpha \right)\rangle & \;\;\;=\;\;\; & N\left(\alpha \right)\left(-\sin\alpha {|+-\rangle }_{aa}+{|-\rangle }_{a}⊗\left\{{\cos\alpha \;|-\rangle }_{a}-\sin\alpha \;{|+\rangle }_{a}\right\}\right)\\ & \;\;\;=\;\;\; & N\left(\alpha \right)\left(-\sin\alpha {|+-\rangle }_{aa}+{|--\rangle }_{ab}\right), \end{array}$$

which shows that $|\psi_{H}\left(\alpha \right)\rangle$ has no |−+〉 component in the ab-basis (property 2).

Finally, starting from Equation (18) (but we could also have started from Equation (19)) and using Equation (8), the transformation from the *b*- to the *a*-basis, we can write $|\psi_{H}\left(\alpha \right)\rangle$ in the bb-basis:

(20) $$\begin{array}{ccc} |\psi_{H}\left(\alpha \right)\rangle & \;\;\;=\;\;\; & {N\left(\alpha \right)(\;|-\rangle }_{b}⊗\left\{{\;\sin\alpha \;|+\rangle }_{b}+\cos\alpha \;{|-\rangle }_{b}\right\}\\ & & \;-\sin\alpha \;\left\{{\;\sin\alpha \;|+\rangle }_{b}+\cos\alpha \;{|-\rangle }_{b}\right\}⊗\left\{{\;\cos\alpha \;|+\rangle }_{b}-\sin\alpha \;{|-\rangle }_{b}\right\})\\ & \;\;\;=\;\;\; & N\left(\alpha \right)(\;-\sin^{2}\;\alpha \cos\alpha {|++\rangle }_{bb}\;+\;\sin^{3}\;\alpha \;{|+-\rangle }_{bb}\\ & & \;+\;\sin^{3}\;\alpha {|-+\rangle }_{bb}\;+\;\cos\alpha \;\left(1+\sin^{2}\alpha \right){|--\rangle }_{bb}). \end{array}$$

This shows that $|\psi_{H}\left(\alpha \right)\rangle$ has both |++〉 and |+−〉 components in the bb-basis (property 4).

To construct a correlation array for the results of Alice and Bob peeling pairs of bananas in the Hardy state, we need $|\psi_{H}\left(\alpha \right)\rangle$ in the aa-, ab-, ba- and bb-basis. Equations (16) and (20) give the state in the aa- and bb-basis, respectively. Starting from Equations (18) and (19), we find the state in the ba- and ab-basis, respectively:

(21) $$\begin{array}{ccc} |\psi_{H}\left(\alpha \right)\rangle & \;\;\;=\;\;\; & N\left(\alpha \right)\left({|--\rangle }_{ba}-\sin\alpha \;\left\{\sin\alpha {|+\rangle }_{b}+\cos\alpha {|-\rangle }_{b}\right\}⊗{|+\rangle }_{a}\right)\\ & \;\;\;=\;\;\; & N\left(\alpha \right)\left({|--\rangle }_{ba}\;-\;\sin^{2}\;\alpha {|++\rangle }_{ba}\;-\;\sin\alpha \cos\alpha {|-+\rangle }_{ba}\right) \end{array}$$

(22) $$\begin{array}{ccc} |\psi_{H}\left(\alpha \right)\rangle & \;\;\;=\;\;\; & N\left(\alpha \right)\left(-\sin\alpha {|+\rangle }_{a}⊗\left\{\sin\alpha {|+\rangle }_{b}+\cos\alpha {|-\rangle }_{b}\right\}+{|--\rangle }_{ab}\right)\\ & \;\;\;=\;\;\; & N\left(\alpha \right)\left(-\sin^{2}\;\alpha {|++\rangle }_{ab}\;-\;\sin\alpha \cos\alpha {|+-\rangle }_{ab}\;+\;{|--\rangle }_{ab}\right) \end{array}$$

**Figure 9:**
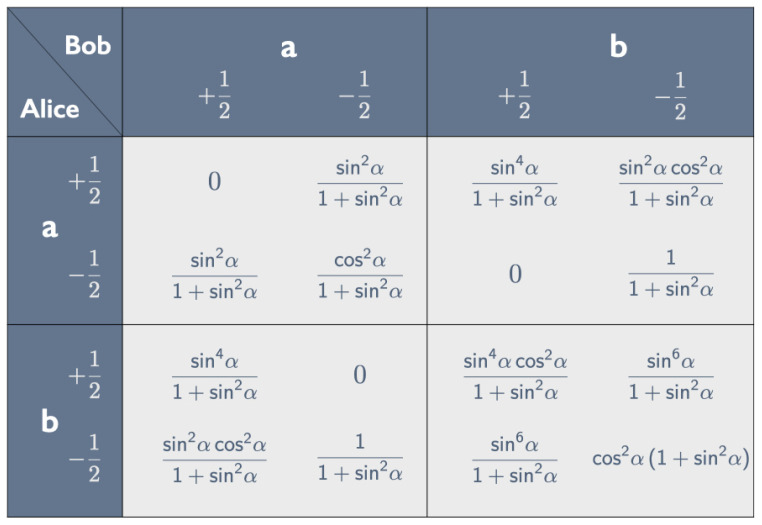
Correlation array for the tastes $\pm \frac{1}{2}$ of pairs of bananas in the Hardy state in Equations (16)–(22), both peeled *a* or *b* by Alice and Bob.

Using the Born rule, we can read off all probabilities entering into the correlation array in Figure 9 from Equations (16) and (20)–(22). One readily checks that (i) in each of the four cells the four entries sum to 1 and (ii) in each row and column the sum of the first two entries is equal to the sum of the last two (though verifying this for the last row involves some tedious algebra). Property (ii) guarantees that the correlation is non-signaling: the marginal probabilities $\mathrm{Pr}(A_{a_{\pm }})$ and $\mathrm{Pr}(A_{b_{\pm }})$ for Alice do not depend on the peeling chosen by Bob and vice versa (cf. [8], pp. 25–26).^6^

We can read the properties of Hardy states listed in Section 3.1 (with $a^{\prime }=a$ and $b^{\prime }=b$) directly off the correlation array in Figure 9. Pr(+−|ba)=0 gives the conditional $A_{b_{+}}\to B_{a_{+}}$ (property 1). Pr(−+|ab)=0 likewise gives the conditional $B_{b_{+}}\to A_{a_{+}}$ (property 2). The aa and bb cells show that the composite conditional is false: $\left(A_{b_{+}}\&\;B_{b_{+}}\right)↛\left(B_{a_{+}}\&\;A_{a_{+}}\right)$. Pr(++|bb)≠0 means that we can have $A_{b_{+}}\&\;B_{b_{+}}$ (property 4); Pr(++|aa)=0 means we cannot have $B_{a_{+}}\&\;A_{a_{+}}$ (property 3).^7^

We cannot simulate this correlation array with one of our raffles because a raffle that gives the 0s in the aa, ab and ba cells must also give a 0 for the ++ entry in the bb cell (cf. the ticket in Figure 8). Finding one instance (or a few to allow for experimental error) of Alice and Bob both finding + when peeling *b* would thus rule out a local hidden-variable theory capable of producing this correlation array.

### 3.3. Hardy States between Maximally Entangled and Product States

The Hardy states $|\psi_{H}\left(\alpha \right)\rangle$ in Equations (16) and (20)–(22) result in the broken arrow in Equation (15) unless α=0 or α=π/2. What happens in those two cases?

For α=0, N(α)=1 and Equations (16) and (18)–(20) reduce to

(23) $$|\psi_{H}\left(0\right)\rangle =|--\rangle$$

in all four bases (aa, ab, ba and bb). The state thus becomes a product state (a property independent of the basis we choose) and the correlations it gives rise to can easily be simulated with one of our raffles.

For α=π/2, $N=1/\sqrt{2}$ and Equations (16) and (20) reduce to

(24) $$|\psi_{H}(\frac{\pi }{2}\rangle =-\frac{1}{\sqrt{2}}\;\left({|+-\rangle }_{aa}+{|-+\rangle }_{aa}\right)=\frac{1}{\sqrt{2}}\;\left({|+-\rangle }_{bb}+{|-+\rangle }_{bb}\right).$$

This is a maximally entangled state (cf. the singlet state in Equation (7)). That the expansion in the aa-basis differs by a minus sign from the expansion in the bb basis reflects that, if α=π/2, ${|+\rangle }_{b}={|-\rangle }_{a}$ and ${|-\rangle }_{b}=-{|+\rangle }_{a}$. It may sound paradoxical that we can simulate the correlations for this maximally entangled state whereas for the non-maximally entangled Hardy states we cannot. Remember, however, that for $\alpha =\varphi_{ab}/2=\pi /2$, the peeling directions *a* and *b* are exactly opposite. In this case (as when α=0 and the peeling directions are the same) we can easily simulate the correlations with one of our raffles.

Kwiat and Hardy [16] consider the special case that $\cos\alpha =\sqrt{2/5}$ and $\sin\alpha =\sqrt{3/5}$, which means that α≈51°. In that case (see Equation (17)),

(25) $$N\left(\alpha \right)\;\sin\alpha =\frac{\sin\alpha }{\sqrt{1+\sin^{2}\;\alpha }}=\sqrt{\frac{3}{8}},\;N\left(\alpha \right)\;\cos\alpha =\frac{\cos\alpha }{\sqrt{1+\sin^{2}\;\alpha }}=\frac{1}{2},$$

and Equation (16) becomes:^8^

(26) $$|\psi_{H}\left(\alpha \approx 51{}^{\circ}\right)\rangle =-\sqrt{\frac{3}{8}}{|+-\rangle }_{aa}+\frac{1}{2}\;{|--\rangle }_{aa}-\sqrt{\frac{3}{8}}\;{|-+\rangle }_{aa}.$$

The Born rule tells us that the probability of Alice and Bob both finding + when both are peeling *b* is equal to the square of the coefficient of |++〉 of $|\psi_{H}\left(\alpha \right)\rangle$ in the bb-basis. For α≈51°, this coefficient is (see Equation (20)):

(27) $$-\frac{\sin^{2}\;\alpha \cos\alpha }{\sqrt{1+\sin^{2}\;\alpha }}=-\frac{\frac{3}{5}\cdot \sqrt{\frac{2}{5}}}{\sqrt{\frac{8}{5}}}=-\frac{3}{5\cdot 2}.$$

Hence Pr(++|bb)=0.09 ([16], p. 34). As Mermin ([17], p. 885) notes, this is “only a shade [≈0.0002] less than the maximum possible” for the square of the expression on the left-hand side of Equation (27). Mermin ([17], p. 884) gives this maximum as ${\left(2/\left(1+\sqrt{5}\right)\right)}^{5}$; Hardy ([2], p. 1667) as $\frac{1}{2}\left(5\sqrt{5}-11\right)$.

This is as far as we will take our analysis of the Hardy family of states. In the next two sections, we scrutinize the intimately related Hardy–Unruh family more closely, especially the dependence of the correlations generated by a branch of this family on the angle α parametrizing this branch.

## 4. Hardy–Unruh States

Inspired by Hardy, Unruh [4] cooked up a family of states providing an even more striking example than Hardy [2] and Kwiat and Hardy [16] of the apparent breakdown of basic logic in quantum mechanics (see Equation (2)). Since the Unruh family turns out to be the same as the Hardy family, we call these states Hardy–Unruh rather than Unruh states. Our discussion in this section mirrors but will be more general than our discussion in Section 3.

### 4.1. Hardy–Unruh Chain of Conditionals

Hardy–Unruh states have four special properties that translate into corresponding properties of the correlations between the tastes found by Alice, peeling *a* or *b*, and Bob, peeling $a^{\prime }$ or $b^{\prime }$, which can but do not have to be the same as *a* and *b*:

1. There is no |+−〉 component in the $ab^{\prime }$-basis. Thus, if Alice peels *a* and finds +, Bob will also find + when he peels $b^{\prime }$. Schematically: $A_{a_{+}}\to B_{b_{+}^{\prime }}$.
2. There is no |−+〉 component in the $bb^{\prime }$-basis. Thus, if Bob peels $b^{\prime }$ and finds +, Alice will also find + when she peels *b*. Schematically: $B_{b_{+}^{\prime }}\to A_{b_{+}}$.
3. There is no |+−〉 component in the $ba^{\prime }$-basis. Thus, if Alice peels *b* and finds +, Bob will also find + when he peels $a^{\prime }$. Schematically: $A_{b_{+}}\to B_{a_{+}^{\prime }}$.
4. There is both a |++〉 and a |+−〉 component in the $aa^{\prime }$-basis. Thus, if Alice peels *a* and finds +, Bob might find + or − when he peels $a^{\prime }$. Schematically: it is possible to have $A_{a_{+}}\;$ and $\;\;B_{a_{-}^{\prime }}$.

**Figure 10:**
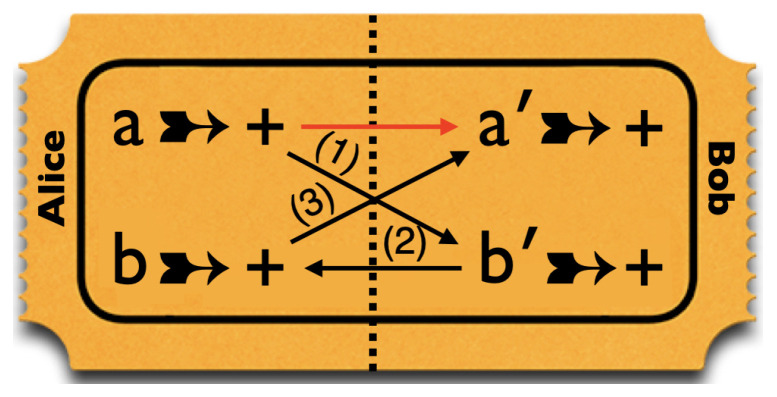
Conflicting demands on the design of a ticket for a raffle simulating the correlations found in measurements on Hardy–Unruh states.

These four properties place contradictory demands on the design of tickets for a raffle simulating these correlations. This is illustrated in Figure 10. Because of the conditionals in 1–3, a ticket with + for *a*, must have + for all four entries. However, property 4 requires our raffle to contain at least *some* tickets with three +s (for *a*, $b^{\prime }$ and *b*) and one − (for $a^{\prime }$).

As Figure 10 illustrates, the conditionals expressing properties 1–3 can be combined into the chain of conditionals

(28) $$A_{a_{+}}\to B_{b_{+}^{\prime }}\to A_{b_{+}}\to B_{a_{+}^{\prime }}.$$

Yet $A_{a_{+}}↛B_{a_{+}^{\prime }}$: it is possible for the antecedent of this conditional to be true and the consequent to be false (property 4). As with the broken arrow in the Hardy case (cf. Equation (15)), quantum mechanics avoids the problem by not allowing truth values to be assigned simultaneously to $A_{a_{+}}$ and $A_{b_{+}}$ or to $B_{a_{+}^{\prime }}$ and $B_{b_{+}^{\prime }}$. The same banana cannot be peeled and tasted twice.

### 4.2. Constructing Hardy–Unruh States

Our construction of the family of Hardy–Unruh states follows the same pattern as our construction of a branch of the family of Hardy states in Equations (16)–(20). We start by making sure that the state has property 2, i.e., that it has no |−+〉 component in the $bb^{\prime }$-basis:

(29) $$|\psi_{HU}\left(u,v,w\right)\rangle =N\left(u{|++\rangle }_{bb^{\prime }}-v{|+-\rangle }_{bb^{\prime }}-w{|--\rangle }_{bb^{\prime }}\right),$$

where *u*, *v* and *w* are arbitrary complex numbers and the normalization factor is given by:

(30) $$N\equiv \frac{1}{\sqrt{{\left|u\right|}^{2}+{\left|v\right|}^{2}+{\left|w\right|}^{2}}}.$$

This shows how generic these Hardy–Unruh states are. We can construct them by starting from a state orthogonal to any two-particle state that can be written in the form |−+〉 in an orthonormal basis $\left\{{|\pm \rangle }_{b}⊗{|\pm \rangle }_{b^{\prime }}\right\}$ of eigenvectors for some pair of peelings *b* and $b^{\prime }$ for Alice and Bob. This is as true for Hardy states as for Hardy–Unruh states.

As we did in Equations (18) and (19), we can group the three terms on the right-hand side of Equation (29) in two different ways:

(31) $$\begin{array}{ccc} |\psi_{HU}\left(u,v,w\right)\rangle & \;\;\;=\;\;\; & N\left({|+\rangle }_{b}⊗\left\{u{|+\rangle }_{b^{\prime }}-v{|-\rangle }_{b^{\prime }}\right\}-w{|--\rangle }_{bb^{\prime }}\right) \end{array}$$

(32) $$\begin{array}{ccc} & \;= & N\left(u{|++\rangle }_{bb^{\prime }}-\left\{v{|+\rangle }_{b}+w{|-\rangle }_{b}\right\}⊗{|-\rangle }_{b^{\prime }}\right) \end{array}$$

Now choose peeling *a* to go with peeling *b* such that the corresponding eigenvectors are:

(33) $$\begin{array}{ccc} {|-\rangle }_{a} & \;\;\;=\;\;\; & \frac{1}{\sqrt{{\left|v\right|}^{2}+{\left|w\right|}^{2}}}\;\left(v{|+\rangle }_{b}+w{|-\rangle }_{b}\right)\\ {|+\rangle }_{a} & \;\;\;=\;\;\; & \frac{1}{\sqrt{{\left|v\right|}^{2}+{\left|w\right|}^{2}}}\;\left(\bar{w}{|+\rangle }_{b}-\bar{v}{|-\rangle }_{b}\right) \end{array}$$

(where bars denote complex conjugates); and choose peeling $a^{\prime }$ to go with peeling $b^{\prime }$ such that the corresponding eigenvectors are:

(34) $$\begin{array}{ccc} {|+\rangle }_{a^{\prime }} & \;\;\;=\;\;\; & \frac{1}{\sqrt{{\left|u\right|}^{2}+{\left|v\right|}^{2}}}\;\left(u{|+\rangle }_{b^{\prime }}-v{|-\rangle }_{b^{\prime }}\right)\\ {|-\rangle }_{a^{\prime }} & \;\;\;=\;\;\; & \frac{1}{\sqrt{{\left|u\right|}^{2}+{\left|v\right|}^{2}}}\;\left(\bar{v}{|+\rangle }_{b^{\prime }}+\bar{u}{|-\rangle }_{b^{\prime }}\right). \end{array}$$

If $\left\{{|\pm \rangle }_{b}\right\}$ and $\left\{{|\pm \rangle }_{b^{\prime }}\right\}$ are orthonormal bases, then $\left\{{|\pm \rangle }_{a}\right\}$ and $\left\{{|\pm \rangle }_{a^{\prime }}\right\}$ are too.

Using Equations (33) and (34), we can write Equations (31) and (32) as

(35) $$\begin{array}{ccc} |\psi_{HU}\left(u,v,w\right)\rangle & \;\;\;=\;\;\; & N\left(\sqrt{{\left|u\right|}^{2}+{\left|v\right|}^{2}}\;{|++\rangle }_{ba^{\prime }}\;-\;w{|--\rangle }_{bb^{\prime }}\right), \end{array}$$

(36) $$\begin{array}{ccc} & \;= & N\left(u{|++\rangle }_{bb^{\prime }}\;-\;\sqrt{{\left|v\right|}^{2}+{\left|w\right|}^{2}}\;{|--\rangle }_{ab^{\prime }}\right). \end{array}$$

Equations (35) and (36) show that $|\psi_{HU}\left(u,v,w\right)\rangle$ has no |+−〉 component in either the $ba^{\prime }$- or the $ab^{\prime }$-basis (properties 1 and 3). Finally, using Equations (33) and (34) to write $|\psi_{HU}\left(u,v,w\right)\rangle$ in the $aa^{\prime }$-basis, one can verify that $|\psi_{HU}\left(u,v,w\right)\rangle$ has both a |++〉 and a |+−〉 component in the $aa^{\prime }$-basis (property 4).

We only verify this last property for the branch of the family we are focusing on in the rest of this paper. The chain in Equation (28) already leads to a broken arrow if Alice and Bob use the same peelings *a* and *b*. In that case, $v=\bar{v}$ and $u=\bar{w}$ in Equations (33) and (34). We take *u* and *w* to be real as well and set:

(37) u=w=cosα,v=sinα,

where, as before, 0<α<π/2 is half the angle $\varphi_{ab}$ between the peeling directions *a* and *b*. With this choice for (u,v,w), Equation (29) becomes:

(38) $$|\psi_{HU}\left(\alpha \right)\rangle =N\left(\alpha \right)\left(\cos\alpha {|++\rangle }_{bb}-\sin\alpha \;{|+-\rangle }_{bb}-\cos\alpha \;{|--\rangle }_{bb}\right),$$

with the normalization factor (cf. Equation (30))

(39) $$N\left(\alpha \right)=\frac{1}{\sqrt{1+\cos^{2}\;\alpha }}.$$

Note the similarity to the Hardy state in Equation (16). Like $|\psi_{HU}\left(\alpha \right)\rangle$, $|\psi_{H}\left(\alpha \right)\rangle$ corresponds to a more general state, $|\psi_{H}\left(u,v,w\right)\rangle$, of the same form as $|\psi_{HU}\left(u,v,w\right)\rangle$ in Equation (29).

With the values for (u,v,w) in Equation (37), Equations (33) and (34) both reduce to Equation (8) for the transformation from the *b*- to the *a*-basis. Using the inverse transformation, Equation (9), and substituting the values of *u*, *v* and *w* in Equation (37) into Equation (35), we find $|\psi_{HU}\left(\alpha \right)\rangle$ in the ba-basis:

(40) $$\begin{array}{ccc} |\psi_{HU}\left(\alpha \right)\rangle & \;\;\;=\;\;\; & N\left(\alpha \right)\left({|++\rangle }_{ba}-\cos\alpha {|-\rangle }_{b}⊗\left(\;-\sin\alpha {|+\rangle }_{a}+\cos\alpha {|-\rangle }_{a}\right)\right)\\ & \;\;\;=\;\;\; & N\left(\alpha \right)\left({|++\rangle }_{ba}+\cos\alpha \sin\alpha {|-+\rangle }_{ba}-\cos^{2}\;\alpha {|--\rangle }_{ba}\right). \end{array}$$

Equation (36) similarly allows us to find $|\psi_{HU}\left(\alpha \right)\rangle$ in the ab-basis:

(41) $$\begin{array}{ccc} |\psi_{HU}\left(\alpha \right)\rangle & \;\;\;=\;\;\; & N\left(\alpha \right)\left(\cos\alpha \left({\;\cos\alpha \;|+\rangle }_{a}+\sin\alpha \;{|-\rangle }_{a}\right)⊗{|+\rangle }_{b}-{|--\rangle }_{ab}\right)\\ & \;\;\;=\;\;\; & N\left(\alpha \right)\left(\cos^{2}\;\alpha {|++\rangle }_{ab}+\cos\alpha \sin\alpha {|-+\rangle }_{ab}-{|--\rangle }_{ab}\right). \end{array}$$

Equations (40) and (41) confirm that $|\psi_{HU}\left(\alpha \right)\rangle$ has no |+−〉 components in the ba- and ab-basis.

Finally, starting from Equation (36)—but we could have started from Equation (35) instead—we find $|\psi_{HU}\left(\alpha \right)\rangle$ in the aa-basis:

(42) $$\begin{array}{ccc} |\psi_{HU}\left(\alpha \right)\rangle & \;\;\;=\;\;\; & N\left(\alpha \right)\left(\cos\alpha {|++\rangle }_{bb}-{|--\rangle }_{ab}\right)\\ & \;\;\;=\;\;\; & N\left(\alpha \right)(\cos\alpha \;\left\{\cos\alpha {|+\rangle }_{a}+\sin\alpha {|-\rangle }_{a}\right\}⊗\left\{\cos\alpha {|+\rangle }_{a}+\sin\alpha {|-\rangle }_{a}\right\}\\ & & \;-\;{|-\rangle }_{a}⊗\left\{\cos\alpha {|-\rangle }_{a}-\sin\alpha {|+\rangle }_{a}\right\})\\ & \;\;\;=\;\;\; & N\left(\alpha \right)(\cos^{3}\;\alpha {|++\rangle }_{aa}+\cos^{2}\;\alpha \sin\alpha {|+-\rangle }_{aa}\\ & & \;+\;\sin\alpha \left(1+\cos^{2}\;\alpha \right){|-+\rangle }_{aa}-\cos^{3}\;\alpha {|--\rangle }_{aa}). \end{array}$$

So $|\psi_{HU}\left(\alpha \right)\rangle$ has both |++〉 and |+−〉 components in the aa-basis (property 4).

**Figure 11:**
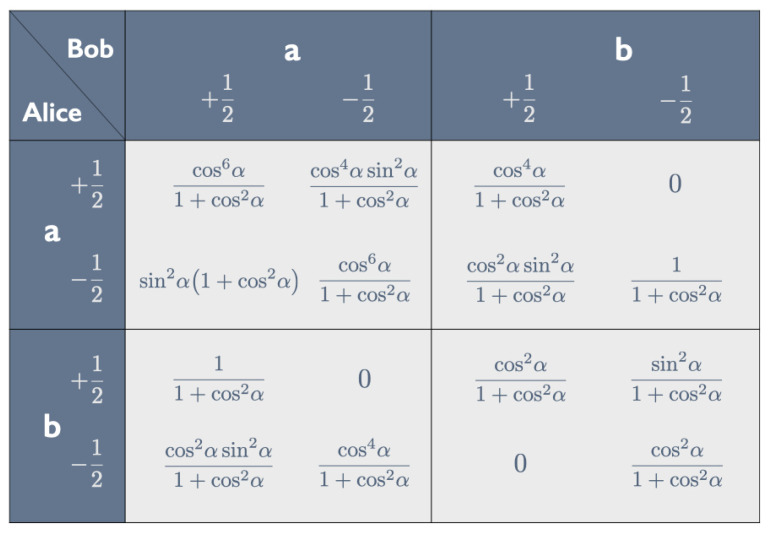
Correlation array for the tastes $\pm \frac{1}{2}$ of pairs of bananas in the Hardy–Unruh state in Equations (38)–(43), both peeled *a* or *b* by Alice and Bob.

We can use Equations (38)–(43) for $|\psi_{HU}\left(\alpha \right)\rangle$ in the aa-, ab-, ba- and bb-bases to construct the correlation array in Figure 11.

As with the correlation array for the Hardy state $|\psi_{H}\left(\alpha \right)\rangle$ in Figure 9, we can read properties 1–4 listed in Section 4.1 (with $a^{\prime }=a$ and $b^{\prime }=b$) of the Hardy–Unruh state $|\psi_{HU}\left(\alpha \right)\rangle$ directly off the correlation array in Figure 11. Pr(+−|ab)=Pr(−+|bb)=Pr(+−|ba)=0 translates into the conditionals $A_{a_{+}}\to B_{b_{+}}$, $B_{b_{+}}\to A_{b_{+}}$ and $A_{b_{+}}\to B_{a_{+}}$ (properties 1–3). Pr(+−|aa)≠0 results in the broken arrow: $A_{a_{+}}↛B_{a_{+}}$.

Relabeling peelings and tastes for Alice and Bob and replacing α by $\frac{\pi }{2}-\alpha$, we can turn the correlation array in Figure 11 for the Hardy–Unruh state $|\psi_{HU}\left(\alpha \right)\rangle$ into the correlation array in Figure 9 for the Hardy state $|\psi_{H}\left(\frac{\pi }{2}-\alpha \right)\rangle$. Specifically, we need to make four changes in these correlation arrays to turn one into the other:

- Switch sinα and cosα.
- Change $\left(a_{\pm },b_{\pm }\right)$ to $\left(b_{\pm },a_{\mp }\right)$ for Alice.
- Change $\left(a_{\pm },b_{\pm }\right)$ to $\left(b_{\mp },a_{\pm }\right)$ for Bob.
- Switch rows and columns to get back to the standard format with labels in the order $\left(a_{+},a_{-},b_{+},b_{-}\right)$ for both Alice and Bob.

As we have seen, the correlation arrays in Figure 9 and Figure 11 capture the defining properties of the Hardy and Hardy–Unruh states listed in Section 3.1 and Section 4.1, respectively. That one can be obtained from the other through the simple expedient of relabeling rows and columns and switching sines and cosines shows that these states are all members of one and the same family.

We cannot simulate the correlation array in Figure 11 with one of our raffles because a raffle that gives the 0s in the ab, ba and bb cells will also give a 0 for the +− entry in the aa cell (cf. the ticket in Figure 10). The +− entry in the aa cell of the correlation array for this Hardy–Unruh state becomes the ++ entry in the bb cell of the correlation array for the corresponding Hardy state. This is the entry that prevents us from simulating the correlation array in Figure 9 for this Hardy state. Any raffle that gives the 0s in the aa, ab and ba cells must give a 0 for the ++ entry in the bb cell (cf. the ticket in Figure 8).

### 4.3. Hardy–Unruh States between Maximally Entangled and Product States

From the correlation array in Figure 11 we can read off that

(43) $$\frac{\mathrm{Pr}(+\;-\;|aa)}{\mathrm{Pr}(+\;+\;|aa)}=\tan^{2}\;\alpha .$$

As α approaches π/2, this ratio grows without bound and the apparent clash with basic logic becomes particularly severe. The chain of conditionals $A_{a_{+}}\to B_{b_{+}}\to A_{b_{+}}\to B_{a_{+}}$ suggests that if Alice and Bob both peel *a* and Alice finds +, Bob should find + as well. Yet, for that peeling combination and for α close to π/2, Bob will almost always find − instead!

If α=π/2, Equations (38)–(43) for $|\psi_{HU}\left(\alpha \right)\rangle$ reduce to:

(44) $$|\psi_{HU}\left(\frac{\pi }{2}\right)\rangle =-{|+-\rangle }_{bb}={|++\rangle }_{ba}=-{|--\rangle }_{ab}={|-+\rangle }_{aa},$$

which is a product state. Thus, we have the paradoxical situation that the apparent clash with ordinary logic gets *worse* as α approaches π/2 but *disappears* when α=π/2! As Unruh ([4], p. 4) observes: “the closer the state is to a product state, a completely un-entangled state, the lower is the probability that if *A* then *D*” (where Unruh’s *A* and *D* are our $A_{a_{+}}$ and $B_{a_{+}}$).

On closer inspection, this discontinuity is only apparent. From the correlation array in Figure 11 we read off that

(45) $$\mathrm{Pr}\left(+\;-\;|aa\right)=\frac{\cos^{4}\;\alpha \sin^{2}\;\alpha }{1+\cos^{2}\;\alpha }.$$

This probability steadily decreases as α approaches π/2 and vanishes for α=π/2. In fact, as both the correlation array in Figure 11 and Equation (44) show, if α approaches π/2 and Alice and Bob both peel *a*, the outcome is almost certainly −+.

Inspection of Figure 4 tells us that, if α=π/2, ${|+\rangle }_{b}={|-\rangle }_{a}$ and ${|-\rangle }_{b}=-{|+\rangle }_{a}$ (this explains the minus signs in Equation (44)). In other words, the operators representing ‘taste when peeled *a*${}^{\prime }$ and ‘taste when peeled *b*${}^{\prime }$ have the same set of eigenvectors. These operators thus commute, in which case the correlations found in measurements on this state can easily be simulated classically (e.g., with one of our raffles). What this means physically becomes clear if we substitute spin-$\frac{1}{2}$ particles for our bananas for a moment. If $\varphi_{ab}=2\alpha =\pi$, the directions *a* and *b* are exactly opposite to one another. Spin up/down in the *a* direction then becomes spin down/up in the *b* direction. The operators representing those observables obviously commute. In fact, we move from one to the other simply by relabeling eigenvectors and eigenvalues.

Since $|\psi_{HU}\left(\frac{\pi }{2}\right)\rangle ={|-+\rangle }_{aa}={|--\rangle }_{ab}$ (see Equation (44)), it is impossible for Alice to peel *a* and find + if α=π/2. This can also be read off the correlation array in Figure 11: all entries in the first row vanish for α=π/2, which means that $\mathrm{Pr}(A_{a_{+}})=0$. Hence, for α=π/2, there is (1) no broken arrow and (2) no problem designing a raffle to simulate the quantum correlations:

1. Since its antecedent is false, the conditional $A_{a_{+}}\to B_{a_{+}}$ is vacuously true and perfectly compatible with the chain of conditionals $A_{a_{+}}\to B_{b_{+}}\to A_{b_{+}}\to B_{a_{+}}$ in Equation (28).
2. Our raffle will have no tickets with + for *a* on Alice’s side, so we avoid the problem with the design of tickets brought out in Figure 8.

If α=0, Equations (38) and (43) for the Hardy–Unruh state $|\psi_{HU}\left(\alpha \right)\rangle$ reduce to

(46) $$|\psi_{HU}\left(0\right)\rangle =\frac{1}{\sqrt{2}}\left({|+-\rangle }_{bb}-{|-+\rangle }_{bb}\right)=\frac{1}{\sqrt{2}}\left({|+-\rangle }_{aa}-{|-+\rangle }_{aa}\right),$$

which is just the maximally entangled singlet state in Equation (7). Yet, there is not even an apparent clash with basic logic and no problem simulating the experiment with one of our raffles. As in the case of the Hardy state $|\psi_{H}\left(\alpha \right)\rangle$, which becomes maximally entangled if α=π/2 (see Equation (24)), this is because the peeling directions *a* and *b* coincide if $\alpha =\varphi_{ab}/2=0$. The tastes of pairs of bananas in the singlet state only exhibit correlations that we cannot simulate with any of our raffles if Alice and Bob get to choose between *different* peeling directions.

## 5. Geometrical Representation of the Correlations Found with Hardy–Unruh States

What can we say about the local polytope L and the quantum convex set Q for the Hardy–Unruh setup (cf. Figure 7)?

**Figure 12:**
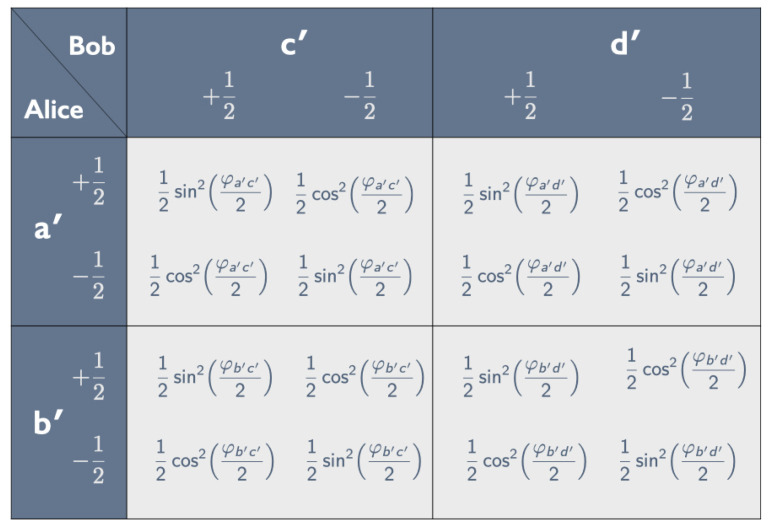
Correlation array for the tastes $\pm \frac{1}{2}$ of pairs of bananas in the singlet state (see Equation (7)), one of them peeled $a^{\prime }$ or $b^{\prime }$ by Alice, the other peeled $c^{\prime }$ and $d^{\prime }$ by Bob.

To answer this question, we start by comparing the correlation array in Figure 11 for the tastes of pairs of bananas, peeled *a* or *b*, in the state $|\psi_{HU}\left(\alpha \right)\rangle$ in Equations (38)–(43) (the Hardy–Unruh setup) to the correlation array in Figure 12 for the tastes of pairs of bananas, one peeled $a^{\prime }$ or $b^{\prime }$, the other peeled $c^{\prime }$ or $d^{\prime }$, in the state $|\psi_{\mathrm{singlet}}\rangle$ in Equation (7) (the CHSH setup).

The correlation array for the CHSH setup consists of four cells of the form shown in Figure 5 and can be fully characterized by four correlation coefficients (see Equation (11)):

(47) $$\begin{array}{ccc} \chi_{a^{\prime }c^{\prime }}=-\cos\varphi_{a^{\prime }c^{\prime }}, & \; & \chi_{a^{\prime }d^{\prime }}=-\cos\varphi_{a^{\prime }d^{\prime }},\\ \chi_{b^{\prime }c^{\prime }}=-\cos\varphi_{b^{\prime }c^{\prime }}, & \; & \chi_{b^{\prime }d^{\prime }}=-\cos\varphi_{b^{\prime }d^{\prime }}. \end{array}$$

The local polytope for this setup is given by the CHSH inequality and three similar pairs of inequalities ([8], pp. 160–161, Equations (5.4)–(5.7)):

(48) $$\begin{array}{c} -2\;\leq \;\chi_{a^{\prime }c^{\prime }}\;+\;\chi_{a^{\prime }d^{\prime }}\;+\;\chi_{b^{\prime }c^{\prime }}\;-\;\chi_{b^{\prime }d^{\prime }}\;\leq 2,\\ -2\;\leq \;-\chi_{a^{\prime }c^{\prime }}\;+\;\chi_{a^{\prime }d^{\prime }}\;-\;\chi_{b^{\prime }c^{\prime }}\;-\;\chi_{b^{\prime }d^{\prime }}\;\leq 2,\\ -2\;\leq \;\chi_{a^{\prime }c^{\prime }}\;-\;\chi_{a^{\prime }d^{\prime }}\;-\;\chi_{b^{\prime }c^{\prime }}\;-\;\chi_{b^{\prime }d^{\prime }}\;\leq 2,\\ -2\;\leq \;-\chi_{a^{\prime }c^{\prime }}\;-\;\chi_{a^{\prime }d^{\prime }}\;+\;\chi_{b^{\prime }c^{\prime }}\;-\;\chi_{b^{\prime }d^{\prime }}\;\leq 2. \end{array}$$

These inequalities can be found in the same way as the pair in Equation (12) for the Mermin setup ([8], pp. 157–159: Figure 5.1 shows the raffle tickets for the CHSH setup, Table 5.1 lists the χ values for these tickets).

The quantum convex set for the CHSH setup is given by a non-linear inequality, first obtained by Landau [18], that follows from the straightforward generalization of the elliptope inequality in Equation (14) if Alice and Bob have four rather than three different peelings to choose from (see [8], p. 166, Equation (5.30), with *a*, *b*, $a^{\prime }$ and $b^{\prime }$ relabeled $a^{\prime }$, $b^{\prime }$, $c^{\prime }$ and $d^{\prime }$):

(49) $$|\chi_{a^{\prime }c^{\prime }}\chi_{b^{\prime }c^{\prime }}-\chi_{a^{\prime }d^{\prime }}\chi_{b^{\prime }d^{\prime }}|\leq \sqrt{1-\chi_{a^{\prime }c^{\prime }}^{2}}\sqrt{1-\chi_{b^{\prime }c^{\prime }}^{2}}+\sqrt{1-\chi_{a^{\prime }d^{\prime }}^{2}}\sqrt{1-\chi_{b^{\prime }d^{\prime }}^{2}}.$$

To use these inequalities for the Hardy–Unruh setup we need to modify the setup somewhat. The problem is that Equations (48) and (49) are derived for balanced variables, i.e., their two possible values are each other’s opposite and equiprobable (see Section 2). This guarantees that their expectation values vanish, which greatly simplifies the expressions for standard deviations and correlation coefficients (see Equations (3) and (5)). While the variables measured by Alice and Bob in the Hardy–Unruh setup have opposite values, their expectation values do not vanish, as these two values are not equiprobable.

We therefore introduce new variables that *are* balanced but have the same covariances as the original ones. The correlations between these new balanced variables for a modified Hardy–Unruh setup can be simulated by a CHSH setup with appropriately chosen peeling directions.^9^ Moreover, the modification preserves an important property of the correlation array for the Hardy–Unruh setup in Figure 11: the ab and ba cells are identical. Hence, we only need three χ parameters to characterize the correlation array for the CHSH setup with which we can simulate the correlations found in the modified Hardy–Unruh setup. This means that the local polytope and the quantum convex set for the modified Hardy–Unruh setup—like those for the Mermin setup (see Figure 7)—can be pictured in three dimensions.

**Figure 13:**
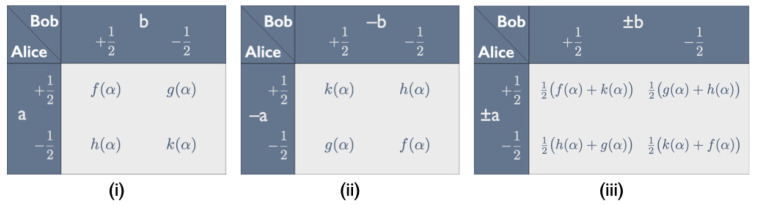
Constructing balanced variables for the Hardy–Unruh setup. The figure shows a cell in the correlation array for Alice and Bob—peeling *a* and *b*, respectively—recording (**i**) the tastes of their bananas, (**ii**) minus those tastes and (**iii**) the tastes in even and minus the tastes in odd runs. The functions f(α), g(α), h(α), k(α) can be read off the correlation array in Figure 11.

We introduce the new balanced variables for the modified Hardy–Unruh setup in two steps. The three panels in Figure 13 illustrate the process for the ab cell. First, we imagine that Alice and Bob, still choosing between peelings *a* and *b*, record the *opposite* of the taste of their bananas. The correlation array for this experiment is obtained by switching the two entries on the diagonal and the two entries on the skew diagonal in each cell of the correlation array in Figure 11 (see panel (ii) in Figure 13 for the ab cell). This obviously flips the signs of the expectation values but does not affect the covariances. As we saw in Equation (4), in each cell, the covariance is equal to $\frac{1}{4}$ times the sum of the two entries on the diagonal minus $\frac{1}{4}$ times the sum of the two entries on the skew diagonal. As these sums stay the same, so do the covariances.

Next, we imagine that Alice and Bob, still choosing between peelings *a* and *b*, record the taste of their bananas in even runs and the *opposite* of the taste in odd runs. We obtain the correlation array for this experiment by taking, for all 16 entries, the straight average of the entries in the correlation arrays for the even and the odd runs (see panel (iii) in Figure 13 for the ab cell). The four covariances are the same in all runs so the covariances for this combined correlation array will still be the same as for the original correlation array in Figure 11. However, by having Alice and Bob alternate between recording the taste and recording minus the taste of their bananas, we ensure that the variables they measure are balanced.

Panel (iii) in Figure 13 shows this for the ab cell, but it is true for all four cells of the combined correlation array. Both entries on the diagonal are the average of the two entries on the diagonal in the original correlation array, and both entries on the skew diagonal are the average of the two entries on the skew diagonal in the original correlation array. Hence, in each cell, the sum of the two entries in each row and in each column gives $\frac{1}{2}$ times the sum of all four probabilities in that cell. The entries in each row and in each column of each cell therefore sum to $\frac{1}{2}$, which means that the variables measured by Alice and Bob when they alternate between recording the taste and minus the taste of their bananas are indeed balanced.

In each cell of the correlation array for the balanced Hardy–Unruh setup, as we call it, the two entries on the diagonal and the two entries on the skew diagonal can be set equal to $\frac{1}{2}$ times the square of, respectively, the sine and the cosine of some angle. Since two of its four cells are identical, the correlation array for the balanced Hardy–Unruh setup can thus be fully characterized by three angles. Identifying these angles with half the angles $\varphi_{a^{\prime }c^{\prime }}$, $\varphi_{a^{\prime }d^{\prime }}=\varphi_{b^{\prime }c^{\prime }}$ and $\varphi_{b^{\prime }d^{\prime }}$ between the peeling directions $a^{\prime }$, $b^{\prime }$, $c^{\prime }$ and $d^{\prime }$, we can cast this correlation array in the form of the correlation array for the CHSH setup in Figure 12.

The standard deviations for the variables in this correlation array are all $\frac{1}{4}$, so the three correlation coefficients characterizing it are given by

(50) $$\begin{array}{c} \begin{array}{c} \chi_{a^{\prime }c^{\prime }}\left(\alpha \right)=4\langle A_{a^{\prime }}B_{c^{\prime }}\rangle =4\langle A_{a}B_{a}\rangle \;,\\ \chi_{a^{\prime }d^{\prime }}\left(\alpha \right)=\chi_{b^{\prime }c^{\prime }}\left(\alpha \right)=4\langle A_{a^{\prime }}B_{d^{\prime }}\rangle =4\langle A_{b^{\prime }}B_{c^{\prime }}\rangle =4\langle A_{a}B_{b}\rangle =4\langle A_{b}B_{a}\rangle \;,\\ \chi_{b^{\prime }d^{\prime }}\left(\alpha \right)=4\langle A_{b^{\prime }}B_{d^{\prime }}\rangle =4\langle A_{b}B_{b}\rangle \;. \end{array} \end{array}$$

where we used that the covariances for this CHSH setup are the same as those for the original Hardy–Unruh setup.

**Figure 14:**
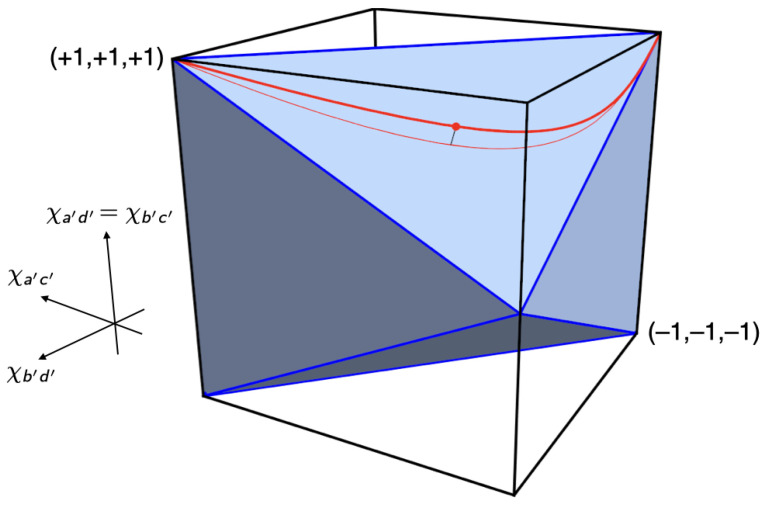
Local polytope L for the CHSH setup with two identical correlation coefficients. The red curve between two of the vertices of L represents the correlations found in the balanced Hardy–Unruh setup for states $|\psi_{HU}\left(\alpha \right)\rangle$ with $0\leq \alpha \leq \frac{\pi }{2}$. The figure also shows the projection of this curve onto a facet of L and the point where the distance between the curve and the facet is maximal.

**Figure 15:**
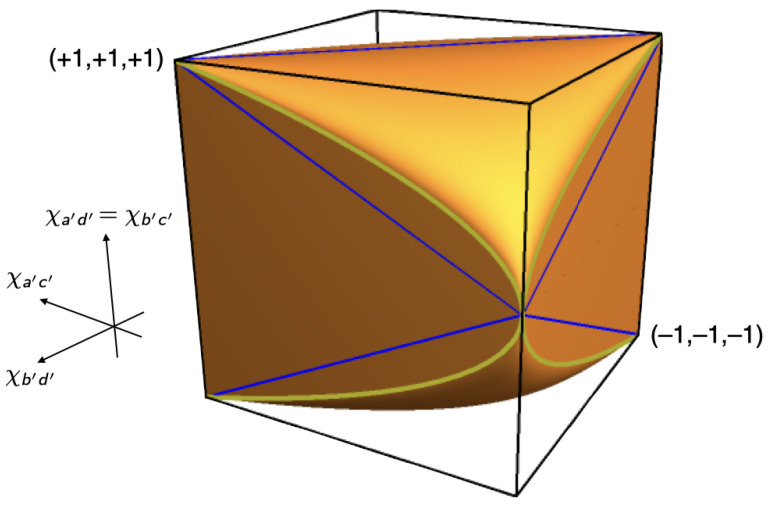
Quantum convex set Q for the CHSH setup with two identical correlation coefficients.

Figure 14 and Figure 15 show the local polytope L and the quantum convex set Q for the subclass of correlations found in the CHSH setup if two of its four correlation coefficients are identical.^10^ We obtain the inequalities defining L and Q in this case by setting $\chi_{a^{\prime }d^{\prime }}=\chi_{b^{\prime }c^{\prime }}$ in Equations (48) and (49). We created Figure 14 and Figure 15 by feeding the resulting inequalities into Mathematica. Note the similarity of these figures to Figure 7 for the Mermin setup. In both cases, Q is an inflated version of L. This ‘inflation’ corresponds to the *pushout* operation in Le et al. ([13], pp. 10–11) and was first found by Masanes [21].

**Figure 16:**
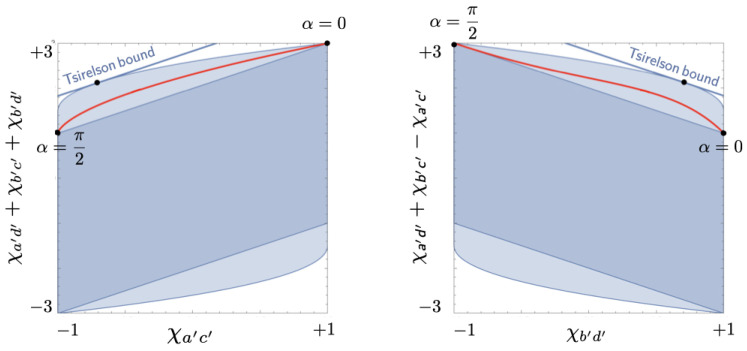
Projections of the local polytope L (the dark blue parallelograms) and the quantum convex set Q (L plus the light blue extensions) for the CHSH setup with two identical correlation coefficients. The red curve represents the projection onto the same plane of the curve representing the correlations found in the balanced Hardy–Unruh setup for the states $|\psi_{HU}\left(\alpha \right)\rangle$ in Equations (38)–(43) with $0\leq \alpha \leq \frac{\pi }{2}$.

The values of the correlation coefficients in Equation (50) parameterize the curve shown in Figure 14 and Figure 16 representing the correlations found between the values of the balanced variables measured on the state $|\psi_{HU}\left(\alpha \right)\rangle$ for $0\leq \alpha \leq \frac{\pi }{2}$ in our balanced Hardy–Unruh setup.

We can compute the covariances on the right-hand side of Equation (50) for these correlation coefficients with the help of the correlation array in Figure 11 (cf. Equation (4)):

(51) $$\begin{array}{c} \langle A_{a}B_{a}\rangle =\frac{1}{4}\cdot \frac{2\cos^{6}\;\alpha -\sin^{2}\;\alpha \;{\left(1+\cos^{2}\;\alpha \right)}^{2}-\cos^{4}\;\alpha \sin^{2}\;\alpha }{1+\cos^{2}\;\alpha },\\ \langle A_{a}B_{b}\rangle =\langle A_{b}B_{a}\rangle =\frac{1}{4}\cdot \frac{\cos^{4}\;\alpha +1-\cos^{2}\;\alpha \sin^{2}\;\alpha }{1+\cos^{2}\;\alpha },\\ \langle A_{b}B_{b}\rangle =\frac{1}{4}\cdot \frac{2\cos^{2}\;\alpha -\sin^{2}\;\alpha }{1+\cos^{2}\;\alpha }. \end{array}$$

Multiplying these expressions by 4 and feeding them into Mathematica, we found the curve in Figure 14 and Figure 16.^11^ These figures clearly show that the correlations found with the state $|\psi_{HU}\left(\alpha \right)\rangle$ are outside the local polytope. As one readily verifies, using Equations (50) and (51), they violate the third pair of CHSH-type inequalities in Equation (48):

(52) $$\chi_{a^{\prime }c^{\prime }}-\chi_{a^{\prime }d^{\prime }}-\chi_{b^{\prime }c^{\prime }}-\chi_{b^{\prime }d^{\prime }}=-\;2-\frac{4\cos^{4}\;\alpha \sin^{2}\;\alpha }{1+\cos^{2}\;\alpha }.$$

The second term on the right-hand side makes the left-hand side smaller than −2. Comparison with Equation (45) shows that this term is equal to 4 times the probability Pr(+−|aa) of the outcome responsible for the broken arrow found with the state $|\psi_{HU}\left(\alpha \right)\rangle$. As the following argument will show, this is no coincidence.

Let *A* and *B* represent the tastes found by Alice and Bob for some combination of peelings. Let Pr(±±) represent the probabilities of the four possible combinations of tastes. Solving four linear equations for these four probabilities, we can express them in terms of the expectation values and the covariance of *A* and *B*.^12^ Normalization gives us the first of these four equations:

(53) Pr(++)+Pr(+−)+Pr(−+)+Pr(−−)=1;

the expectation values of *A* and *B* give us two more:

(54) $$\begin{array}{ccc} \langle A\rangle & \;\;\;=\;\;\; & \frac{1}{2}\left(\mathrm{Pr}(++)+\mathrm{Pr}(+-)-\mathrm{Pr}(-+)-\mathrm{Pr}(--)\right), \end{array}$$

(55) $$\begin{array}{ccc} \langle B\rangle & \;\;\;=\;\;\; & \frac{1}{2}\left(\mathrm{Pr}(++)-\mathrm{Pr}(+-)+\mathrm{Pr}(-+)-\mathrm{Pr}(--)\right); \end{array}$$

and the covariance of *A* and *B* gives us the last one:

(56) $$\langle AB\rangle =\frac{1}{4}\left(\mathrm{Pr}(++)-\mathrm{Pr}(+-)-\mathrm{Pr}(-+)+\mathrm{Pr}(--)\right).$$

Multiplying Equations (54) and (55) by 2 and Equation (56) by 4 and solving the resulting equations for the four probabilities, we find:

(57) $$\begin{array}{ccc} \mathrm{Pr}(++) & \;\;\;=\;\;\; & \frac{1}{4}+\frac{1}{2}\;\langle A\rangle +\frac{1}{2}\;\langle B\rangle +\langle AB\rangle ,\\ \mathrm{Pr}(+-) & \;\;\;=\;\;\; & \frac{1}{4}+\frac{1}{2}\;\langle A\rangle -\frac{1}{2}\;\langle B\rangle -\langle AB\rangle ,\\ \mathrm{Pr}(-+) & \;\;\;=\;\;\; & \frac{1}{4}-\frac{1}{2}\;\langle A\rangle +\frac{1}{2}\;\langle B\rangle -\langle AB\rangle ,\\ \mathrm{Pr}(--) & \;\;\;=\;\;\; & \frac{1}{4}-\frac{1}{2}\;\langle A\rangle -\frac{1}{2}\;\langle B\rangle +\langle AB\rangle . \end{array}$$

If the expectation values vanish, the probabilities are equal to $\frac{1}{4}$ plus or minus the covariance. Setting the covariance equal to $4\chi_{ab}$, we recover the entries in the correlation array in Figure 2. At this point, however, we are interested in the case that the expectation values do *not* vanish.

Consider the probabilities that are 0 in the ab, ba and bb cells of the correlation array in Figure 11 and the non-vanishing probability in the aa cell that is responsible for the broken arrow in the Hardy–Unruh chain. This gives us the following four equations:

(58) $$\begin{array}{c} \mathrm{Pr}\left(+\;-\;|aa\right)=\frac{1}{4}+\frac{1}{2}\;\langle A_{a}\rangle -\frac{1}{2}\;\langle B_{a}\rangle -\langle A_{a}B_{a}\rangle ,\\ 0=\mathrm{Pr}\left(+\;-\;|ab\right)=\frac{1}{4}+\frac{1}{2}\;\langle A_{a}\rangle -\frac{1}{2}\;\langle B_{b}\rangle -\langle A_{a}B_{b}\rangle ,\\ 0=\mathrm{Pr}\left(+\;-\;|ba\right)=\frac{1}{4}+\frac{1}{2}\;\langle A_{b}\rangle -\frac{1}{2}\;\langle B_{a}\rangle -\langle A_{b}B_{a}\rangle ,\\ 0=\mathrm{Pr}\left(-\;+\;|bb\right)=\frac{1}{4}-\frac{1}{2}\;\langle A_{b}\rangle +\frac{1}{2}\;\langle B_{b}\rangle -\langle A_{b}B_{b}\rangle . \end{array}$$

If the last three are subtracted from the first, the expectation values all cancel and we are left with:

(59) $$\mathrm{Pr}\left(+\;-\;|aa\right)=-\frac{1}{2}-\langle A_{a}B_{a}\rangle +\langle A_{a}B_{b}\rangle +\langle A_{b}B_{a}\rangle +\langle A_{a}B_{b}\rangle .$$

Multiplying both sides by 4 and regrouping terms, we can rewrite this as:

(60) $$4\langle A_{a}B_{a}\rangle -4\langle A_{a}B_{b}\rangle -4\langle A_{b}B_{a}\rangle -4\langle A_{a}B_{b}\rangle =-2-4\mathrm{Pr}(+\;-\;|aa).$$

Using Equation (50) to replace 4 times the covariances by the corresponding correlation coefficients and using Equation (45) for Pr(+−|aa), we recover Equation (52). This shows, to reiterate, that the violation of the corresponding CHSH-type inequality is given by the probability of the outcome responsible for the broken arrow in the Hardy–Unruh chain. The maximum value of this probability is the same as the maximum value of the probability Pr(++|bb) of the outcome responsible for the broken arrow in the Hardy chain (see Equation (27)).

## 6. Conclusions

Our examination of Hardy–Unruh chains has left us with a trifecta of deflating insights. The third is that we cannot claim great originality for the first two. Yet even those for whom they are hardly new will agree, we hope, that our use of the framework of *Understanding Quantum Raffles* [8] helped put these insights in sharper relief. In this short concluding section, we summarize how our analysis in terms of raffle tickets, correlation arrays and their geometrical representation led us to these insights. Our treatment of Hardy–Unruh chains also connects the literature on Hardy’s paradox with the (much more extensive) literature on correlation polytopes (see note 14) and will hopefully contribute to making the latter more widely accessible.

The first insight is that the states giving rise to the broken arrow in Hardy’s chain of conditionals in Equations (1) and (15) are no different from those giving rise to the broken arrow in Unruh’s chain of conditionals in Equations (2) and (28). All these states are part of one large family of non-maximally entangled states (how large can be gleaned from our construction of a generic member in Equations (29)–(36), which simplifies the constructions given by Hardy [2] and Unruh [4]). We exhibited these family ties by constructing correlation arrays for correlations leading to both kinds of broken arrows, the one in Figure 9 for the Hardy states $|\psi_{H}\left(\alpha \right)\rangle$ in Equations (16) and (20)–(22), and the one in Figure 11 for the Hardy–Unruh states $|\psi_{HU}\left(\alpha \right)\rangle$ in Equations (38)–(43). We showed how the defining properties of Hardy and Hardy–Unruh chains of conditionals can be read off these correlation arrays. We then showed that these two correlation arrays differ only in how they are labeled (the peelings *a* and *b*, the tastes + and −, and the angle α parametrizing the states). Although we only did this for members of a specific branch of the Hardy–Unruh family, the same could be done for any family member.

The second insight is that a broken arrow in a Hardy–Unruh chain is equivalent to the violation of some Bell inequality. We showed this (again: for a special branch of the Hardy–Unruh family) by constructing a geometrical representation of the correlation array for the Hardy–Unruh setup (see Figure 14, Figure 15 and Figure 16). What complicated this task is that the two possible values of the variables measured in the Hardy–Unruh setup are not equiprobable. We took care of this problem by slightly modifying the Hardy–Unruh setup. We could then use a special case of the CHSH inequality (and similar inequalities associated with other facets of the local polytope) to characterize the class of correlations in this modified Hardy–Unruh setup allowed by a local hidden-variable theory (i.e., the class of correlations in this setup that can be simulated with one of our raffles). We showed (see Equation (60)) that the term that expresses the violation of one of these CHSH-type inequalities is exactly the same as the expression for the probability of the very outcome that is responsible for the broken arrow in the corresponding Hardy–Unruh chain. This result may not come as a surprise to many readers, but it was still worth proving.

We agree with Mermin ([17], pp. 883–884) that one should not exaggerate the difference between using one single outcome or the statistics of many outcomes as evidence that a correlation is not to be had in a local hidden-variable theory. If, for instance, we want to simulate the correlation array for a Hardy–Unruh setup in Figure 9 or Figure 11 with one of our raffles, the problem is *not* to obtain a non-zero probability for one particular outcome, but to obtain it *while at the same time obtaining zero probabilities for several other outcomes*.^13^ In other words, rather than focusing on individual entries, we need to consider a correlation array as a whole.^14^

Despite being taken down a notch, Hardy–Unruh chains remain valuable. Whereas we usually consider violations of Bell inequalities by correlations found in measurements on maximally entangled states, Hardy–Unruh chains forcefully demonstrate that the slightest amount of entanglement already makes it impossible to simultaneously assign definite values to variables represented by non-commuting operators.

## Figures and Tables

**Table 1:** Values of the anti-correlation coefficients for raffles with just one of the four types of tickets shown in Figure 6.

Ticket	$\chi_{ab}$	$\chi_{ac}$	$\chi_{bc}$
(i)	−1	−1	−1
(ii)	−1	+1	+1
(iii)	+1	−1	+1
(iv)	+1	+1	−1

## Data Availability

Not applicable.

## Notes

1 See Liang, Spekkens and Wiseman [9] for an earlier analysis of the violation of the transitivity of material implication in quantum mechanics noted by Hardy [2]. We are grateful to Yeong-Cherng Liang for drawing our attention to this article.

2 The figures in this section are all based on figures in our book [8]. As in the book (see p. xvii), the two of us focus on pedagogy (Janssen) and polytopes (Janas) and leave the philosophy to our Canadian co-author (see Cuffaro’s paper in this issue: “The Measurement Problem Is a Feature, Not a Bug—Schematising the Observer and the Concept of an Open System on an Informational, or (Neo-)Bohrian, Approach”). Fittingly, the title of our paper comes from a Neil Young song on Buffalo Springfield’s sophomore album. A song with the same title appears on the first solo album of another Canadian artist, Robbie Robertson (1943–2023).

3 As Popescu wrote in his foreword to *Bananaworld*: “The more I think of the bananas, the more I like their use: The two peeling actions [Bub only allowed two diffferent peelings, from the top and from the stem] are complementary and cannot both be implemented on the same banana—once it’s peeled it’s peeled, exactly like two measurements that cannot be simultaneously performed on microscopic particles. And there is only one answer—once it’s eaten it’s eaten. Again, exactly like in quantum mechanics. The magic bananas are a perfect model for what is going on. So much better than the abstract models the physicists use” ([6], p. vi).

4 In our book ([8], pp. 72–73), $\chi_{ab}$ is defined as an *anti*-correlation coefficient. The extra minus sign affects Table 1, Figure 7 and Equations (13) and (14) below.

5 See Goh et al. [11], Chen et al. [12] and Le et al. [13] for interesting recent work in this tradition, to which Tsirelson made important early contributions (see, for instance, [14], p. 3, Figure 2). In a paper from 1987 [15], Tsirelson already noted: “As one can easily see, the set Cor(m,n) of all quantum realized m×n correlation matrices … is a closed, bounded, centrally symmetric, convex body in the space of m×n matrices” (p. 562).

6 This is true for any compound system with a Hilbert space that is the tensor product of the Hilbert spaces of its components. We sketch a simple proof of this property for the situation at hand, which can readily be adapted to the general case. The probability that Alice finds + when she peels *a* and Bob peels *b* can be written as

$$\mathrm{Pr}\left(A_{+}|A_{a},B_{b},\hat{\rho }\right)=\mathrm{Pr}\left(A_{+}\&\;B_{+}|A_{a},B_{b},\hat{\rho }\right)+\mathrm{Pr}\left(A_{+}\&\;B_{-}|A_{a},B_{b},\hat{\rho }\right),$$

where $\hat{\rho }$ is the density operator characterizing the quantum statistical ensemble under consideration. We are considering an ensemble (or *quantum raffle* in our terminology [8]) of pairs of quantum bananas all in the state $|\psi_{HU}\left(\alpha \right)\rangle$, in which case $\hat{\rho }=|\psi_{HU}\left(\alpha \right)\rangle \langle \psi_{HU}\left(\alpha \right)|$, the projection operator onto that state, but the same argument works for any ensemble of pairs of quantum bananas in any state. Using the trace formula for the Born rule for both terms, we can write the right-hand side as

$$\mathrm{Tr}\left(\hat{\rho }\;\left({\hat{P}}_{1_{a_{+}}}\;\;⊗{\hat{P}}_{2_{b_{+}}}\right)\right)+\mathrm{Tr}\left(\hat{\rho }\;\left({\hat{P}}_{1_{a_{+}}}\;\;⊗{\hat{P}}_{2_{b_{-}}}\right)\right).$$

where ${\hat{P}}_{1_{a_{\pm }}}$ and ${\hat{P}}_{2_{b_{\pm }}}$ are the projection operators in the Hilbert spaces of the bananas of Alice (subscript 1) and Bob (subscript 2) onto the eigenvectors in the *a*- and *b*-bases. Using basic properties of the trace operation and the tensor product, we can rewrite this expression as

$$\mathrm{Tr}\left(\hat{\rho }\;\left({\hat{P}}_{1_{a_{+}}}\;\;⊗\left[{\hat{P}}_{2_{b_{+}}}+{\hat{P}}_{2_{b_{-}}}\right]\right)\right).$$

Using the completeness of the *b*-basis to set ${\hat{P}}_{2_{b_{+}}}+{\hat{P}}_{2_{b_{-}}}={\hat{1}}_{2}$, we arrive at

$$\mathrm{Pr}\left(A_{+}|A_{a},B_{b},\hat{\rho }\right)=\mathrm{Tr}\left(\hat{\rho }\;\left({\hat{P}}_{1_{a_{+}}}\;\;⊗{\hat{1}}_{2}\right)\right).$$

Had Bob chosen *a*, we would have arrived at the exact same result, using the completeness of the *a*-basis. This shows that this marginal probability is indeed independent of the peeling Bob chooses.

7 The correlation array in Figure 9—such as the one in Figure 11 below—belongs to ‘Class 3a: Three blocks [cells in our terminology] having one zero each’ in the helpful classification of 2×2 correlation arrays by Chen et al. [12].

8 Kwiat and Hardy present their example in terms of quantum cakes rather than quantum bananas. Our conditions 1–4 are their conditions 2, 2${}^{\prime }$, 3 and 1, respectively ([16], p. 34). Instead of ‘taste when peeled *a*${}^{\prime }$ and ‘taste when peeled *b*${}^{\prime }$ (with values + for yummy and − for nasty), they introduce the variables ‘taste’ (with values *G* and *B* for ‘good’ and ‘bad’) and ‘rising of batter’ (with values for *R* and *N* for ‘risen’ and ‘not risen’). The corresponding orthonormal bases, {|G〉,|B〉} and {|R〉,|N〉}, are related via (cf. Equation (8) for α≈51°):

$$|G\rangle =\sqrt{\frac{2}{5}}\;|R\rangle -\sqrt{\frac{3}{5}}\;|N\rangle ,\;|B\rangle =\sqrt{\frac{3}{5}}\;|R\rangle +\sqrt{\frac{2}{5}}\;|N\rangle .$$

Using the {|G〉,|B〉} basis for the Hilbert space of both quantum cakes, they write the state in Equation (26) as (with *L* and *R* for ‘left’ or ‘Lucien’ and ‘right’ or ‘Ricardo’ instead of our 1 or 2):

$$|\psi \rangle =\frac{1}{2}\;|B_{L}\rangle |B_{R}\rangle -\sqrt{\frac{3}{8}}\;\left[|B_{L}\rangle |G_{R}\rangle +|G_{L}\rangle |B_{R}\rangle \right]$$

(see [16], p. 35, Appendix).

9 Given a correlation array for measurements on any two-particle state, we can find a correlation array with the same covariances (though not the same expectation values) for measurements on the singlet state. This is a direct consequence of theorem 1 in Tsirelson’s 1980 paper ([19], pp. 93–94; for the proof see his 1987 paper [15]). Our introduction of balanced variables for the Hardy–Unruh setup was inspired by the proof of part of this theorem by Avis et al. ([20], p. 7).

10 The depiction of the convex sets in Figure 14 and Figure 15 was also found by Le et al. ([13], p. 9, Figure 2), who moreover extensively review the larger convex sets in the absence of the $\chi_{a^{\prime }d^{\prime }}=\chi_{b^{\prime }c^{\prime }}$ constraint.

11 Both projection plots in Figure 16 are in the spirit of Goh et al. [11]. The convex sets depicted are equivalent (up to a minor change in variables) to those in Figure 5 on p. 8 of their paper. This is not entirely trivial: their Figure 5 depicts the $\chi_{a^{\prime }d^{\prime }}=\chi_{b^{\prime }c^{\prime }}=\chi_{b^{\prime }d^{\prime }}$ section of the relevant convex sets, whereas, for instance, our first plot amounts to a *projection* onto this same hyperplane. However, the non-trivial symmetries for these sets (see Le et al. [13]), in conjunction with their convexity, ensure that Goh et al.’s *section* and our *projection* are identical.

12 These probabilities can also be expressed directly as expectation values of the corresponding operators in the quantum state under consideration. Take, for instance, the operators ${\hat{A}}_{a}$ and ${\hat{B}}_{b}$ representing the variables $A_{a}$, the taste of Alice’s banana when peeled *a*, and $B_{b}$, the taste of Bob’s banana when peeled *b*. We can write the projection operators onto the eigenvectors ${|\pm \rangle }_{a}$ and ${|\pm \rangle }_{b}$ of these operators as:

$${\hat{P}}_{a_{\pm }}=\frac{\hat{1}\pm 2{\hat{A}}_{a}}{2},\;{\hat{P}}_{b_{\pm }}=\frac{\hat{1}\pm 2{\hat{B}}_{b}}{2}.$$

For any ensemble of pairs of bananas (characterized by some density operator $\hat{\rho }$), the probability Pr(+−|ab) is given by the expectation value of the tensor product of these projection operators:

$$\mathrm{Pr}\left(+\;-\;|ab\right)=\langle {\hat{P}}_{1_{a_{+}}}⊗{\hat{P}}_{2_{b_{-}}}\rangle =\frac{1}{4}\;\langle \left({\hat{1}}_{1}+2{\hat{A}}_{a}\right)⊗\left({\hat{1}}_{2}-2{\hat{B}}_{b}\right)\rangle =\frac{1}{4}+\frac{1}{2}\langle {\hat{A}}_{a}\rangle -\frac{1}{2}\langle {\hat{B}}_{b}\rangle -\langle {\hat{A}}_{a}{\hat{B}}_{b}\rangle ,$$

which corresponds to the second line in Equation (57).

13 Mermin [17] further considers whether this problem may be circumvented by supposing these other probabilities are sufficiently small, rather than strictly zero. Relaxing this assumption in Equation (58), the passage to Equation (60) no longer finds that the violation of the CHSH inequality is controlled by Pr(+−|aa) alone. Instead, the relevant quantity is

Pr(+−|aa)−Pr(+−|ab)−Pr(−+|ba)−Pr(−−|bb).

But this quantity is no longer manifestly nonnegative, and so cannot yield an inequality stronger than the CHSH inequality itself (Braun and Choi [22], p. 6). (As stressed by Mermin as well as Braun and Choi, the statement that this quantity is *nevertheless* nonnegative is the Clauser-Horne inequality [23]).

14 For another (admittedly more convoluted) example involving measurements in the Mermin setup on a pair of spin-1 particles in the singlet state, see *Understanding Quantum Raffles* ([8], pp. 136–137).
